# Improving healthcare professionals’ interactions with patients to tackle antimicrobial resistance: a systematic review of interventions, barriers, and facilitators

**DOI:** 10.3389/fpubh.2024.1359790

**Published:** 2024-05-22

**Authors:** Abimbola Ayorinde, Iman Ghosh, Junaid Shaikh, Victoria Adetunji, Anna Brown, Mary Jordan, Ellie Gilham, Daniel Todkill, Diane Ashiru-Oredope

**Affiliations:** ^1^Warwick Medical School, University of Warwick, Coventry, United Kingdom; ^2^UK Health Security Agency, London, United Kingdom; ^3^School of Pharmacy, University of Nottingham, Nottingham, United Kingdom

**Keywords:** antimicrobial resistance, interactions, barriers, facilitators, healthcare professional

## Abstract

**Introduction:**

Antimicrobial resistance (AMR) is a major public health threat. With the growing emphasis on patient-centred care/ shared decision making, it is important for healthcare professionals’ (HCPs) who prescribe, dispense, administer and/or monitor antimicrobials to be adequately equipped to facilitate appropriate antimicrobial use. We systematically identified existing interventions which aim to improve HCPs interaction with patients and examined barriers and facilitators of appropriate the use of such interventions and appropriate antimicrobial use among both HCPs and patientsantimicrobial use while using these interventions.

**Methods:**

We searched MEDLINE, EMBASE, Web of Science, Google Scholar, and internet (via Google search engine). We included primary studies, published in English from 2010 to 2023 [PROSPERO (CRD42023395642)]. The protocol was preregistered with PROSPERO (CRD42023395642). We performed quality assessment using mixed methods appraisal tool. We applied narrative synthesis and used the COM-B (Capability, Opportunity, Motivation -Behaviour) as a theoretical framework for barriers and facilitators at HCP and patient levels.

**Results:**

Of 9,172 citations retrieved from database searches, From 4,979 citations remained after removal of duplicates. We included 59 studies spanning over 13 countries. Interventions often involved multiple components beyond HCPs’ interaction with patients. From 24 studies reporting barriers and facilitators, we identified issues relating to capability (such as, knowledge/understanding about AMR, diagnostic uncertainties, awareness of interventions and forgetfulness); opportunity (such as, time constraint and intervention accessibility) and motivation (such as, patient’s desire for antibiotics and fear of litigation).

**Conclusion:**

The findings of this review should be considered by intervention designers/adopters and policy makers to improve utilisation and effectiveness.

## Introduction

Antimicrobial resistance (AMR) occurs when ‘bacteria, viruses, fungi and parasites change over time and no longer respond to medicines making infections harder to treat and increasing the risk of disease spread, severe illness and death’ (1). Globally, bacterial AMR was estimated to be associated with 4·95 million deaths in the year 2019 (2). This is predicted to increase to 10 million deaths per year by 2050 with a cumulative cost of 100 trillion USD if no action is taken (3). This global catastrophe demands immediate attention.

Healthcare professionals (HCPs) including doctors, nurses, pharmacists, and other licenced individuals trained to prescribe, dispense, administer, and/or monitor antimicrobials are uniquely positioned to reduce AMR. Although regulations regarding prescribing practises vary for different countries (4). There is a growing emphasis on patient-centred care, which encourages shared decision-making between HCPs and patients (5, 6). Research has identified numerous mechanisms that facilitate HCPs in embracing shared decision-making practises, part of which involves enhancing HCPs skills and confidence in engaging patients in decision-making (7). Interventions have been implemented and evaluated with the aim of empowering HCPs to interact effectively with patients about the appropriate use of antimicrobials in different health conditions (8, 9). These interventions encompass a range of approaches, such as communication skills training, patient information leaflets, multicomponent toolkits and point-of-care C reactive protein (CRP) testing, each showing varying success (8). Despite the availability of such interventions, various challenges, such as time constraints and concerns about potential complications, may hinder HCPs and patients from making the right decisions regarding antimicrobial use (8). Recognising and addressing these barriers is crucial for optimising the use of exiting interventions and improving interactions between HCPs and patients to tackle antimicrobial resistance.

A significant aspect of interventions to tackle AMR focus on improving and maintaining individual antimicrobial prescribing and antimicrobial use behaviour, though the wider use of targeted behaviour change interventions is still emerging (10). Many theories of understanding behaviour and behaviour change have been identified to have potential relevance in designing and evaluating public health interventions (11). One of such is the Capability, Opportunity, Motivation, Behaviour (COM-B) model, the core model of behaviour in the Behaviour Change Wheel (BCW) (12). The COM-B model proposed that behaviour is influenced by the interaction of the three components and changing behaviour will involve changing one or more of the three components: capability, opportunity, and motivation (12). Capability refers to psychological and physical capacity of the individual to exhibit the relevant activity/behaviour (12). Capability can be psychological (knowledge or psychological skills, knowledge or stamina) or physical (physical skills, strength or stamina). Opportunity refers to external factors that that make the behaviour possible or prompt the behaviour (12). Opportunity can be physical (that is, opportunity afforded by the environment) or social (opportunity afforded by interpersonal influences, social cues and cultural norms). Motivation includes all cognitive processes that energise and direct the behaviour, which can be automatic (emotion) or reflective (beliefs, intentions) (12). Various primary studies have used the BCW and COM-B model to develop interventions and to understand factors influencing behaviour relating to AMR and infection control (13–16). This includes, for example, development of antibiotic review toolkit (13), understanding how antimicrobial stewardship education and training are implemented (15), understanding hand hygiene (16) among others. BCW and COM-B model are now often used in evidence synthesis to facilitate the identification of areas of improvement and potential interventions (17, 18), By applying the COM-B model to existing studies that explore the barriers and facilitators of utilising the available interventions that aimed at improving HCPs interaction with patients and of appropriate antimicrobial use, we can develop a thorough understanding of areas of improvement and strategies to achieve them.

This review aimed to identify AMR interventions which focus on improving HCPs’ interactions with patients. It also aimed to use the COM-B framework to group the evidence collated concerning the barriers and facilitators associated with the utilisation of such interventions and appropriate antimicrobial use among both HCPs and patients.

## Methods

### Information sources

Between January 31, 2023 and March 27, 2023 we searched electronic databases; MEDLINE All (via Ovid), EMBASE (via Ovid), Science Citation Index (via Web of Science), Social Sciences Citation Index (via Web of Science) and Google Scholar. To identify additional studies and grey literature, we conducted forward, and backward citation searching from eligible studies and searched the internet using Google search engine.

### Search strategy

The search strategy included terms relating to antimicrobial use/prescribing, HCPs and interventions aimed at HCPs interactions with patients, and barriers/facilitators. It used a combination of free text and thesaurus (MeSH/Emtree) terms. Searches were limited to studies published in English Language since 2010 (see search strategies in Appendix 1). Citations were exported into Endnote 20, deduplicated, and then exported onto Rayyan to facilitate screening. Rayyan is an online tool that facilitates title and abstract screening as well as collaboration between reviewers (19). All titles and abstracts were screened by a single reviewer and a random sample of 10% of the citations were double screened by a second reviewer. Full-texts of selected titles were independently screened by two reviewers (AA and IG, JS, and VA). Discrepancies were resolved by discussion between reviewers and when necessary, with a third reviewer and/or the wider team.

### Selection criteria

#### Inclusion criteria

Population: Any HCP involved in antimicrobial prescribing, dispensing and administration.

Intervention: Interventions which focused on HCPs’ interactions with patients including interventions that empower HCPs to have better conversations with patients/public regarding antimicrobial resistance. That is, interventions that are directly involved in HCPs and patients’ interactions during consultation. For example, specific skills training, patient information leaflets, and electronic decision support tools which HCPs may use while having dialogue with patients. These patient interaction components may be standalone interventions or included as a part of intervention with multiple components.

Comparators/controls: Any or none.

Outcome: Barriers and facilitators of appropriate behaviours for the HCPs and patients. For example, patient demand (patient); prescribing when they would prefer not to/giving in to perceived demand (HCPs). HCPs’ and patients’ knowledge, attitudes, beliefs, and behaviour regarding antimicrobial use in relation to the intervention.

Study types: Any primary study design.

Publication date: Only studies published from the year 2010 were included to focus on more current issues.

#### Exclusion criteria

We excluded interventions that do not target HCPs’ interaction with patients, such as public campaigns and interventions that focus solely on educating HCPs without involving direct interface between HCPs and public/patient. We also excluded articles that are not based on original studies such as topical reviews, essays, and expert opinions. We excluded systematic reviews but screened the reference list of related reviews to identify any relevant studies. Studies published before year 2010 and those that are not published in English Language were excluded.

### Data extraction

We designed a data extraction form on Microsoft Excel to extract the relevant information from each study. This includes study ID; country; methods; characteristics of participants; description of intervention; outcome; and influence of patient interaction (barriers, facilitators). We extracted information on the interventions using the Template for Intervention Description and Replication (TIDieR) guideline for clarity and consistency across included studies (20). One reviewer completed the data extraction, and a second reviewer checked the data.

### Quality assessment

We used the mixed methods appraisal tool (MMAT) (21) to assess the quality of included studies. The MMAT covers five study designs (qualitative studies, randomised controlled trials, non-randomised studies, quantitative descriptive studies, and mixed methods studies) and each has five quality criteria with three response options (‘Yes’, ‘No’ or ‘Cannot tell’). One reviewer (AA, IG, JS, or VA) performed quality assessment of all included studies, while a second reviewer independently assessed a subset (17%), resulting in an agreement rate of 93%. The disagreements were resolved by discussion. We calculated the proportion of ‘yes’ for each article to show the proportion of the quality criteria each article met.

### Data synthesis

We synthesised the evidence narratively. We tabulated the intervention characteristics based on TIDieR. For studies which report barriers and facilitators, we used theoretically informed thematic synthesis approach to synthesise findings relating to barriers and facilitators of appropriate behaviours for the HCPs and patients. We used NVivo software to aid this coding process. We used the COM-B as a theoretical framework (12). To do this, one reviewer inductively coded findings from the studies into descriptive themes and the themes were mapped to the relevant COM-B components based on their definitions. Using this theoretical framework helps to facilitate the identification of possible BCW intervention types which may be used to mitigate barriers identified (12).

The protocol was pre-registered with PROSPERO (CRD42023395642) and findings are reported according to PRISMA guidelines (22).

## Results

**Figure 1:**
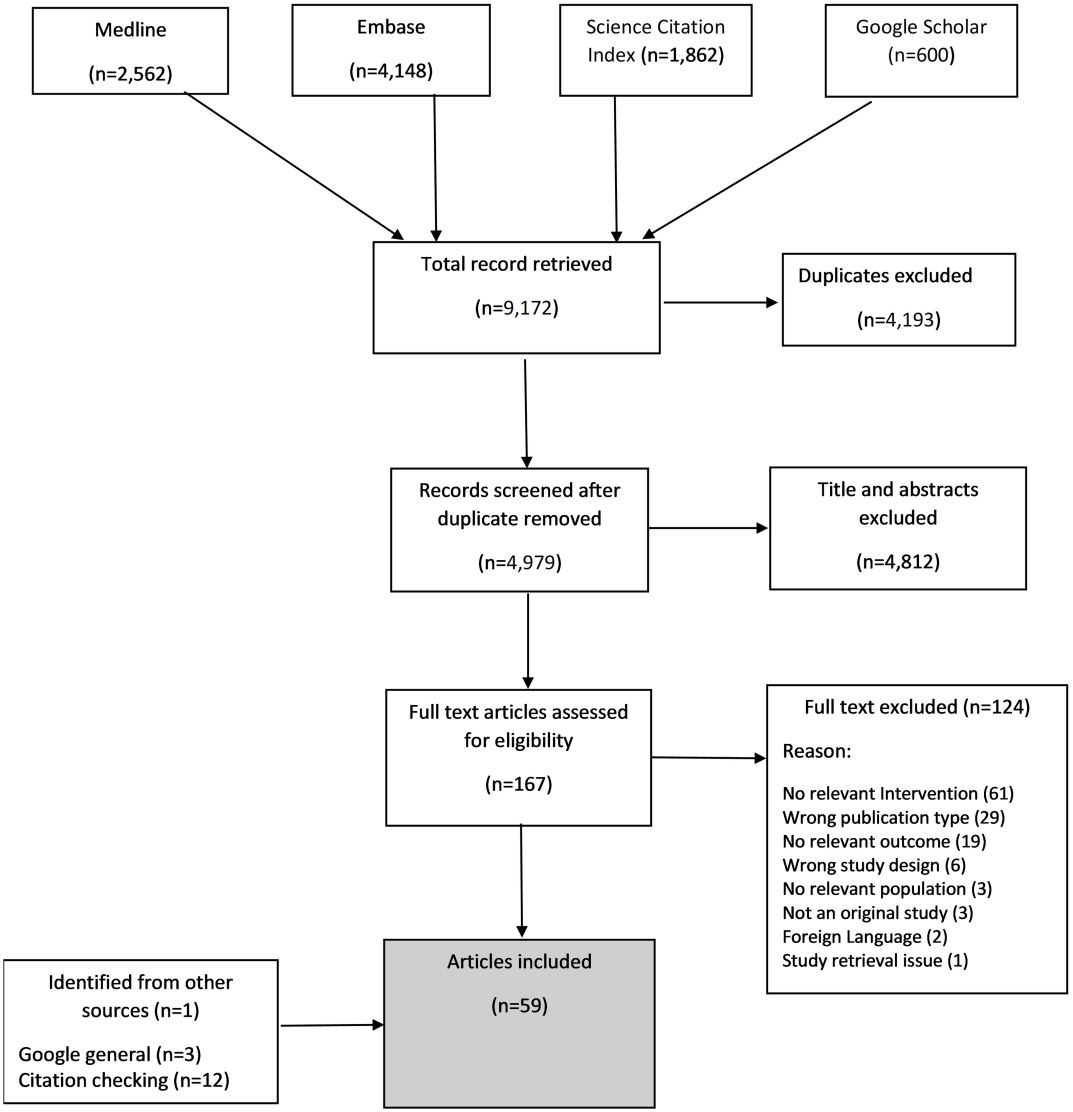
PRISMA flowchart of study selection.

The electronic database search yielded 9,172 citations of which 4,979 remained after removing duplicates. After screening titles and abstracts, we retained 167 studies of which 43 were included (see Appendix 2 for excluded studies). Additional 16 papers were identified from other sources (such as Google search, citation search). In total 59 articles were included in the review and 24 contributed to the synthesis of barriers and facilitators. An overview of the study selection is presented in Figure 1. The characteristics of included studies are presented in Table 1. The studies were conducted across more than 13 countries, with the majority (*n* = 17 studies) conducted in United Kingdom (25, 28, 32, 37, 40, 41, 43, 44, 48–51, 53, 63, 67, 76, 77), followed by 13 studies from the United States (23, 24, 35, 42, 46, 47, 52, 62, 65, 68, 69, 71, 75), six studies from the Netherlands (33, 34, 38, 39, 70, 78), three studies each from Canada (36, 54, 64) and Germany (72–74), two each from Australia (29, 30), Spain (60, 61), Belgium (55, 56), and China (79, 80), and single study each from France (45), Sweden (66), Russian federation (26), and Latvia (57). Five studies recruited participants from multiple countries (27, 31, 58, 59, 81). Most studies (*n* = 46) were conducted in primary care settings, seven were in secondary care, two included both secondary and primary care, three in community pharmacies and one in nursing homes. There were a range of study designs, although most were randomised controlled trials (*n* = 25), followed by quantitative non-randomised (*n* = 12) and qualitative studies (*n* = 10). Most of the HCP-patient interaction was by general practitioners/family physicians/doctors except for three, where explicitly the pharmacist played the significant role (25, 28, 77).

**Table 1:** Characteristics of included studies.

Study ID (Author, Year); Study location	Study design	Participants which health care professionals are involved (number of participants if available)	Intervention; (Patient interactive component)	Are there findings on barriers and facilitators of appropriate behaviours for the healthcare professionals and patients reported?	Study setting
Ackerman, (23); United States	Mixed method including an RCT	All Primary care clinicians (physicians, physician assistants, and nurse practitioners) (55 Clinicians Recruited 29 Completed)	Bespoke anti-microbial stewardship (Patient education brochures)	✓	Primary care
Agency For Healthcare Research and Quality, 2022 (24); United States	Cohort study	Physicians and pharmacists (physicians and pharmacists from 14 acute care hospitals, seven long-term care facilities, and nine ambulatory care practises participated)	The safety programme (Commitment posters and patient handouts)	X	Primary care
Allison, 2020 (25); United Kingdom	Quantitative questionnaire study	Pharmacy staff (pharmacists, pre-registration trainee pharmacists, healthcare counter staff, dispensary staff, technicians, pharmacy manager and pharmacy assistant) (12 pharmacies comprise of 43 pharmacy staff)	The pharmacy antimicrobial stewardship intervention (PAMSI) (An Antibiotic Checklist, AMS reinforcing materials, which included posters, shelf signs, counter mats and prescription bag stickers)	✓	Community pharmacy
Andreeva, 2014 (26); Russian Federation	An open cluster randomised clinical trial	General practitioners (GPs) (HCP: 18)	Bespoke anti-microbial stewardship (CRP testing)	X	Primary care
Anthierens, 2015 (27); Multi-Country	Qualitative study	General practise clinicians (HCPs: 66)	Genomics to combat resistance against antibiotics in community-acquired LRTI in Europe INternet TRaining for antibiOtic use (GRACE INTRO) (Training in communication skills with use of a patient booklet)	✓	Primary care
Ashiru-Oredope, 2020 (28); United Kingdom	A non-blinded cluster randomised control trial	Community pharmacies-Pharmacy staff (182 pharmacies)	The TARGET ‘treating your infection—respiratory tract infection’ (TARGET-TYI-RTI) (TARGET TYI-RTI community pharmacy leaflet)	✓	Community pharmacies
Avent, 2024 (29); Australia	A cluster randomised trial (Quantitative and qualitative component)	GPs (GPs from 27 practises)	General practitioner antimicrobial stewardship programme study (GAPS) (Posters, patient information leaflet)	✓	Primary care
Biezen, 2021 (30); Australia	Qualitative intervention study	VicREN practise HCP (GP, practise nurse) (HCP: eight practises, 14 GPs, one practise nurse)	Bespoke anti-microbial stewardship (Seven patient information sheets)	✓	Primary care
Bjerrum, 2011 (31); Multi-country	Audit Project Odense (APO)	GPs (HCPs 440)	Health Alliance for Prudent Prescribing, Yield and Use of Antimicrobial Drugs in the Treatment of Respiratory Tract Infections (HAPPY AUDIT) (Posters, Brochures and handouts to patients)	X	Primary care
Butler, 2012 (32); United Kingdom	Randomised controlled trial	Clinicians [clinicians from 68 practises (34 each arm)]	Stemming the Tide of Antibiotic Resistance programme (STAR) programme (Video-rich material presenting novel communication skills based on motivational interviewing)	X	Primary care
Cals, 2010 (33) The Netherlands	Randomised controlled trial	Family Physicians (HCPs 32)	C reactive protein (CRP) assistance (Consultation with the nurse and CRP testing)	X	Primary care
Cals, 2013 (34); The Netherlands	A pragmatic, factorial, cluster-randomised controlled trial	Family physicians (40 family physicians from 20 practises)	Bespoke anti-microbial stewardship (Physicians communication skill for cough consultation)	X	Primary care
Chiswell, 2019 (35); United States	A quasi-experimental pretest–post-test design	PC practise staff (HCPs: NR)	Bespoke anti-microbial stewardship (Patient education materials, posters and videos)	X	Primary care
Chung, 2017 (36); Canada	Qualitative study	Stakeholder and paediatric ED providers including ED physicians, nurse practitioners, physician assistants, and residents (HCP: 22 individuals)	Electronic health record–based clinical decision support (EHR CDS) (EHR CDS)	✓	Secondary care
Cross, 2019 (37); United Kingdom	A single-site study	Consultant, trainee grade doctor, pharmacist, nurse and patients (HCPs:175)	Antibiotic Review Kit (ARK) (Patient leaflet)	X	Secondary care
Dekker, 2018 (38); The Netherlands	A cluster randomised controlled trial with measurements before and after	GPs (35 GPs were in control arm and 40 GPs in intervention arm)	Bespoke anti-microbial stewardship (Patient information booklet)	X	Primary care
Dekker, 2019 (39); The Netherlands	Cluster two-arm RCT	GPs (30 GPs)	RAAK (RAtional Antibiotic use Kids) intervention (A written information booklet for parents)	X	Primary care
Eley, 2018 (40); United Kingdom	Nested qualitative study	Practise staff from GP practises (12 practises and 26 general practise staff)	Point of care C reactive protein test (CRP POCT) (CRP testing)	✓	Primary care
Eley, 2020 (41); United Kingdom	Service evaluation	HCPs and GP (43 HCPs, 15 GPs)	TARGET The Treating Your Infection (TYI) (Version 8) (TARGET Treating Your Infection leaflet)	✓	Primary care
Forrest, 2020 (42); United States	Mixed method Plan-Do-Study-Act cycles	Nurse practitioners, physician assistants Practical nurse, and registration staff (HCPs:18)	Bespoke anti-microbial stewardship (Shared decision aids)	✓	Secondary care
Francis, 2013 (43); United Kingdom	Qualitative study	Clinicians (13 Out of 51clinician participated)	Enhancing the Quality of Information-sharing in Primary Care (EQUIP) study (Interactive booklet)	✓	Primary care
Francis, 2020 (44); United Kingdom	RCT, process and economic evaluation	Clinicians (e.g., GPs, nurse practitioners, practise nurses and health-care assistants) (Clinicians from 86 practises)	The PACE (Primary care use of A C-reactive protein point-of-care test to help target antibiotic prescribing to patients with acute Exacerbations of chronic obstructive pulmonary disease who are most likely to benefit) (CRP testing)	✓	Primary care
Giry, 2016 (45); France	A cross-sectional survey	Family physician (HCPs: 283)	Bespoke anti-microbial stewardship (Handing out of a factsheet, Using specific prescription with an educational message for patients)	✓	Primary care
Goggin, 2022 (46); United States	A multisite, parallel group, cluster randomised trial	Clinicians (HCP: 51)	Bespoke anti-microbial stewardship (90s video and information brochure)	X	Primary care
Gonzales, 2013 (47); United States	Three-arm cluster randomised trial	Board certified internal medicine and family practise physicians, nurse practitioners, physician assistants, and registered nurses (HCPs: NR)	Bespoke anti-microbial stewardship (Patient educational brochures and a poster)	X	Primary care
Gulliford, 2014 (48); United Kingdom	RCT	Family practises [HCPs from 50 family practises (each arm)]	VISON (A single-sided patient information sheet)	X	Primary care
Hernandez-Santiago, 2015 (49); United Kingdom	Cohort study	General practises (HCPs: NR)	Bespoke anti-microbial stewardship (Patient information leaflets and posters)	X	Primary care
Hounkpatin, 2021 (50); United Kingdom	Qualitative study	GPs (HCPs:32)	Respiratory tract infections-clinical prediction rules (RTI CPRs) (CRP testing)	✓	Primary care
Huddy, 2016 (51); United Kingdom	Qualitative study	GPs (including those with commissioning experience), biochemists, pharmacists, clinical laboratory scientists and industry representatives (HCP: Stage 1: 11 Invited, 8 Agreed, Stage 2: 24 Invited 10 Attended)	Point of care C reactive protein (POC CRP) (CRP testing)	✓	Primary care
Jenkins, 2013 (52); United States	RCT	Practise staff (HCPs: 46 study group and 34 the control group)	Bespoke anti-microbial stewardship (Patient education materials)	X	Primary care and secondary care
Jones, 2017 (53); United Kingdom	A mixed method study	GP and stakeholders [269 (quant) and 53 (qual)]	Treat Antibiotics Responsibly; Guidance, Education, Tools (TARGET) Antibiotics Toolkit [Patient leaflets (Treating Your Infection)]	✓	Primary care
Legare, 2012 (54); Canada	Multi centre, parallel cluster randomised trial	Family physicians, including physician teachers and residents (12 family practise comprised of 162 family physicians)	DECISION+2 (Decision support tool)	X	Primary care
Lemiengre, 2018 (55); Belgium	A cluster randomised, factorial controlled trial	Family physicians (FPs) (131 FPs from 78 practises Analysed)	Brief intervention to elicit parental concern combined with safety net advice (BISNA) and point of care C reactive protein (POC CRP) (A parent information leaflet)	X	Primary care
Lemiengre, 2018 (56); Belgium	RCT	Family physicians (FPs) [HCP 133 (analysed)]	ERNIE2 trial-point of care C reactive protein (POC CRP) (CRP testing)	X	Primary care
Likopa, 2022 (57); Latvia	RCT	General practises (HCPS 80)	Point of care C reactive protein test (CRP POCT) (Parent information booklets)	X	Primary care
Little, 2019 (58); Multi-country	RCT and audit	Clinicians and nurses (HCPs: 372)	Bespoke anti-microbial stewardship (An interactive patient booklet)	X	Primary care
Little, 2013 (59); Multi-country	A multinational, cluster, randomised, factorial, controlled trial	General practises include clinicians and nurse prescribers (HCP: 259 practises randomised and 228 analysed)	Bespoke anti-microbial stewardship (Training in enhanced communication skills)	X	Primary care
Llor, 2014 (60); Spain	A prospective non-randomised before-and-after study	GP or Physician (HCPs: 235 Full intervention, 97 Partial intervention)	Health Alliance for Prudent Prescribing, Yield and Use of Antimicrobial Drugs in the Treatment of Respiratory Tract Infections (HAPPY AUDIT) (Posters for doctors’ waiting rooms, Brochures and handouts for patients)	X	Primary care
Llor, 2015 (61); Spain	Experimental study	GPs (primary care physicians) (HCPs: 281)	Health Alliance for Prudent Prescribing, Yield and Use of Antimicrobial Drugs in the Treatment of Respiratory Tract Infections (HAPPY AUDIT) (Posters for doctors’ waiting rooms, Brochures and handouts for patients)	X	Primary care
Madaras-Kelly, 2020 (62); United States	A quasi-experimental controlled study	Clinicians from emergency departments and primary care clinics (Approximately 170 clinicians from ED and PCCs)	Bespoke anti-microbial stewardship (Patient educational materials for distribution during visit)	X	Primary care and secondary care
McDermott, 2014 (63); United Kingdom	A mixed method evaluation	GP and practise staff [107 participants (Evaluation), 24 Participants (Qualitative), 83 GPs (Questionnaire)]	Bespoke anti-microbial stewardship (Electronic educational and decision support tools)	✓	Primary care
McIsaac, 2021 (64); Canada	A quasi-experimental pre-and post-study design	Clinicians, pharmacists, and support staff (HCPs: 86)	Bespoke point of care anti-microbial stewardship (Patient education materials)	X	Primary care
Meeker, 2014 (65); United States	Randomised clinical trial	Clinicians (HCPs: 14)	Bespoke anti-microbial stewardship (A posted commitment letter)	X	Primary care
Milos, 2013 (66); Sweden	RCT	Participants from private primary health care centres (PHCCs) (22 PHCCs Comprises of 139 GPs)	Bespoke anti-microbial stewardship (Persuasive communication intervention)	X	Primary care
Mowbray, 2020 (67); United Kingdom	Qualitative study	Medical staff involved in discharging patients (HCPs: NR)	ARK-Hospital intervention-GRACE-INTRO (Patient education leaflet)	✓	Secondary care
Muhia, 2016 (68); United States	A pre-and post-test quality improvement project	Healthcare providers, which consisted of MDs, PAs, NPs, interns and registered nurses (HCPs: 30)	Bespoke anti-microbial stewardship (Patient education material)	x	Primary care
Patel, 2022 (69); United States	Survey	Clinicians (38 response form clinicians)	Bespoke anti-microbial stewardship (Poster and a trifold patient education pamphlet)	X	Secondary care
Peters, 2013 (70); The Netherlands	A prospective case–control study	Primary care staff (Primary care staff from two centres)	Point of care C reactive protein (POC CRP) [CRP testing]	X	Primary care
Pittenger, 2015 (71); United States	A retrospective time series study and cost analysis	Primary care providers (family practise and general internal medicine physicians, nurse practitioners, and physician assistants) [HCPs: 118 (At Seven Sites)]	Bespoke anti-microbial stewardship (Nursing phone care)	X	Primary care
Poss-Doering, 2020 (72) (a); Germany	Qualitative study	Physicians (27 Primary Care Physicians)	Arena (Sustainable reduction of antibiotic-induced antimicrobial resistance) study (E-learning on communication with patients, information material on tablet computer for patients)	✓	Primary care
Poss-Doering, 2020 (73) (b); Germany	RCT nested with in a mixed method study	Healthcare provider team (GPs and MAs) (HCPs from 114 practises)	CHANGE-3 (Converting Habits of Antibiotic Use for Respiratory Tract Infections in German Primary Care) (Thematically focused information and a web-and paper-based public awareness campaign)	✓	Primary care
Poss-Doering, 2020 (74); Germany	A mixed method evaluation	GPs and non-physician health professionals (HCPs: 340)	CHANGE-3 (Converting Habits of Antibiotic Use for Respiratory Tract Infections in German Primary Care) (Educational contents for patients)	✓	Primary care
Sloane, 2020 (75); United States	Two-year quality improvement trial with two arms	Physicians, nurse practitioners, and physician assistants [27 (NH chain 14, Provider group13)]	Antibiotic Stewardship Training and Quality Improvement Intervention (Bespoke) (Information brochure in lay language)	X	Tertiary care (community nursing homes)
Tonkin-Crine, 2023 (76); United Kingdom	A mixed method evaluation	General practises (nine practise comprises of 81 HCPs, 13 HCPs participated in interviews)	Bespoke anti-microbial stewardship-point of care C reactive protein test (POC-CRPT) (Patient leaflets)	✓	Primary care
Tonna, 2020 (77); Scotland, United Kingdom	Qualitative study	Pharmacists, pharmacy students, pharmacy technician and medicine counter assistants (HCPs:28)	Bespoke anti-microbial stewardship (Self-help guide leaflet)	✓	Community pharmacy
van Esch, 2018 (78); The Netherlands	A questionnaire survey	GPs (15 general practises)	Bespoke anti-microbial stewardship [Shared decision making (SDM) Questionnaire]	X	Primary care
Wei, 2017 (79); China	A parallel-group, cluster-randomised controlled trial	Participants from Township hospitals-Doctors (Doctors from 25 Township hospitals)	Bespoke anti-microbial stewardship (Leaflets and a video educating caregivers)	X	Secondary care
Wei, 2019 (80); China	Two-arm, cluster-randomised controlled trial with Mixed method approach	Doctors (doctors from 25 Township Hospitals)	Bespoke anti-microbial stewardship (Leaflets and a video educating caregivers)	✓	Secondary care
Yardley, 2013 (81); Multi-country	A quantitative process study nested within a cluster-randomised controlled trial	GP practises (229 practises and 346 GPs)	Genomics to combat resistance against Antibiotics in Community-acquired LRTI in Europe/INternet Training for Reducing antibiOtic use (GRACE/INTRO) (Patient booklet)	X	Primary care

**Figure 2:**
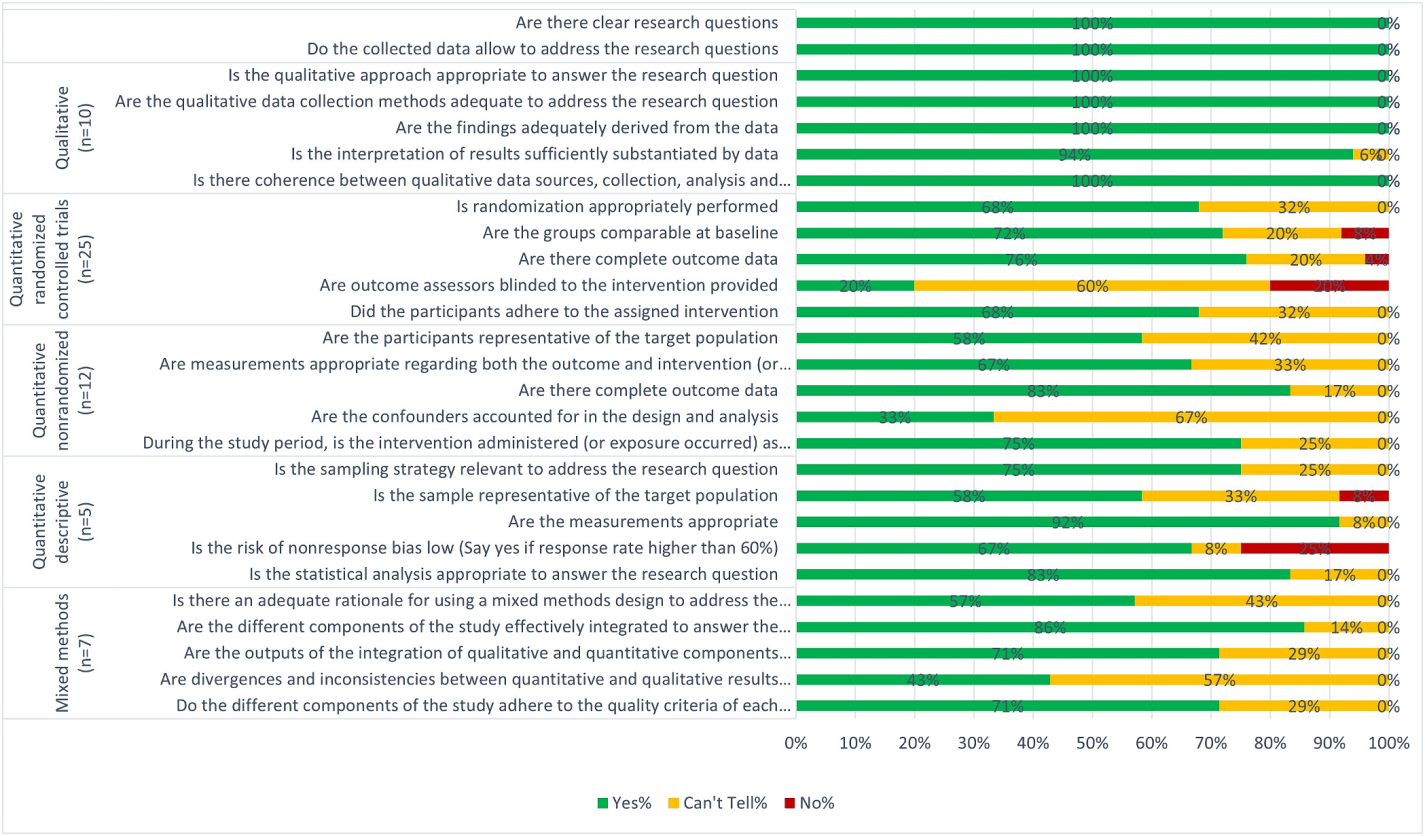
Quality appraisal of included studies.

A summary of the quality of the studies is presented in Figure 2 and details for individual studies are presented in Appendix 3. We consider most of the studies to be of good quality as 15 studies fulfilled 100% of the relevant quality criteria, 19 studies fulfilled between 80–90% and 13 fulfilled 60%. Although the remaining 12 studies fulfilled less than 50%, this was mostly due to not clearly reporting information related to the criteria concerning intervention effectiveness (Figure 2; Appendix 3).

Various types of interventions were evaluated (Appendix 4). Some were established strategies, such as Treat Antibiotics Responsibly; Guidance, Education (TARGET) (28, 41, 53), antibiotics review kit (ARK) (37), Health Alliance for Prudent Prescribing, Yield and Use of Antimicrobial Drugs in the Treatment of Respiratory Tract Infections (HAPPY AUDIT) (60, 61), Genomics to combat Resistance against Antibiotics in Community-acquired LRTI in Europe Internet TRaining for antibiOtic use (GRACE INTRO) (27, 81) and Converting Habits of Antibiotic Use for Respiratory Tract Infections in German Primary Care (CHANGE-3) (72, 74), while some were bespoke antimicrobial stewardship programmes (23, 26, 30, 34, 35, 38, 42, 46, 47, 49, 52, 58, 59, 62–66, 68, 69, 77–80). C-reactive protein point-of-care testing was often reported (26, 27, 33, 34, 40, 50, 51, 56–59, 70, 76, 81). The majority of the studies (*n* = 51) reported that interventions include a patient interactive component in the form of posters, leaflet, videos, interactive decision support tools (Table 1; Appendix 4).

**Figure 3:**
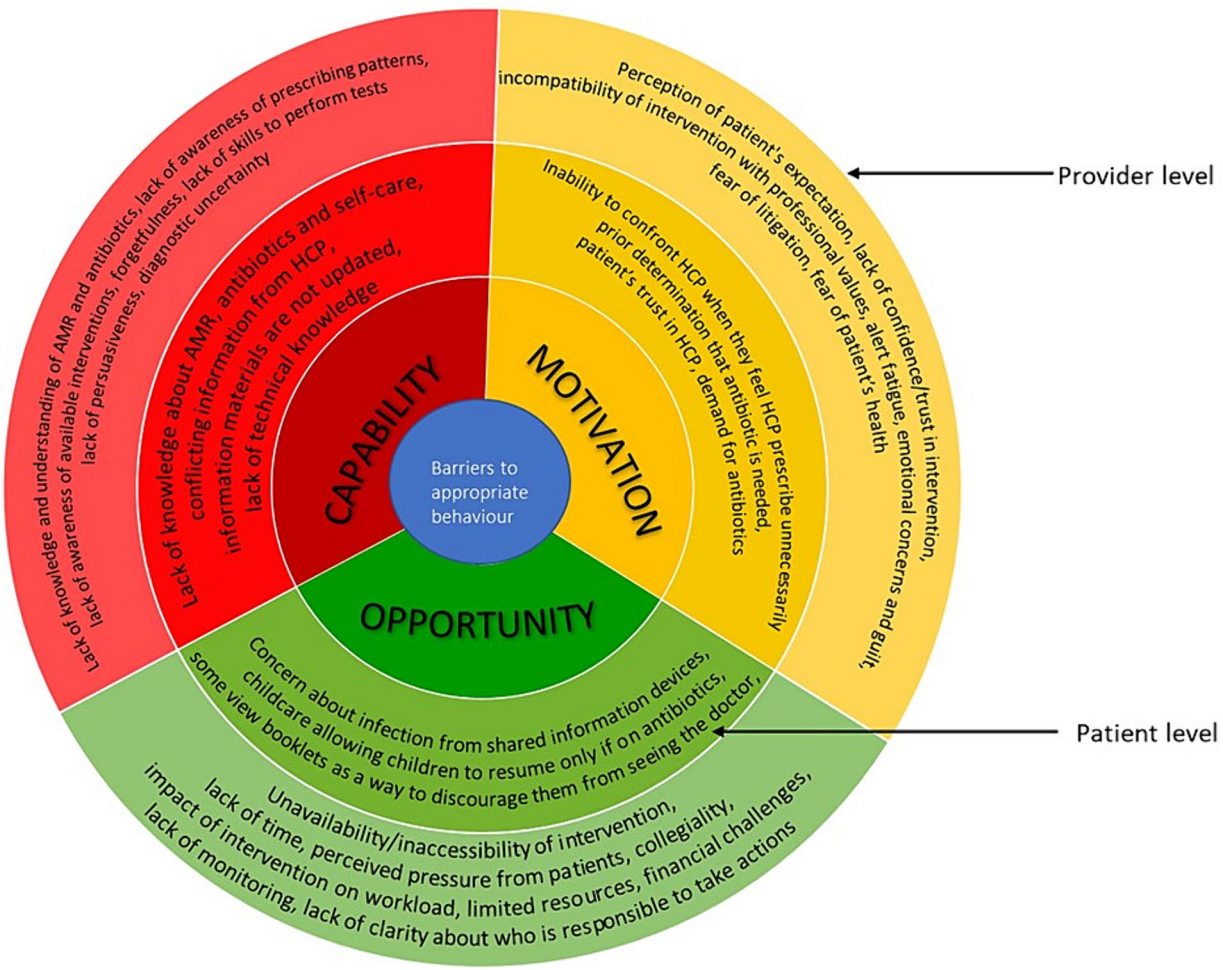
Barriers to appropriate antimicrobial behaviour at healthcare professionals and patient levels mapped on to the COM-B model. The image summarises the barriers identified from included studies. The outer layer refers to the provider level and inner layer refers to the patient level.

**Figure 4:**
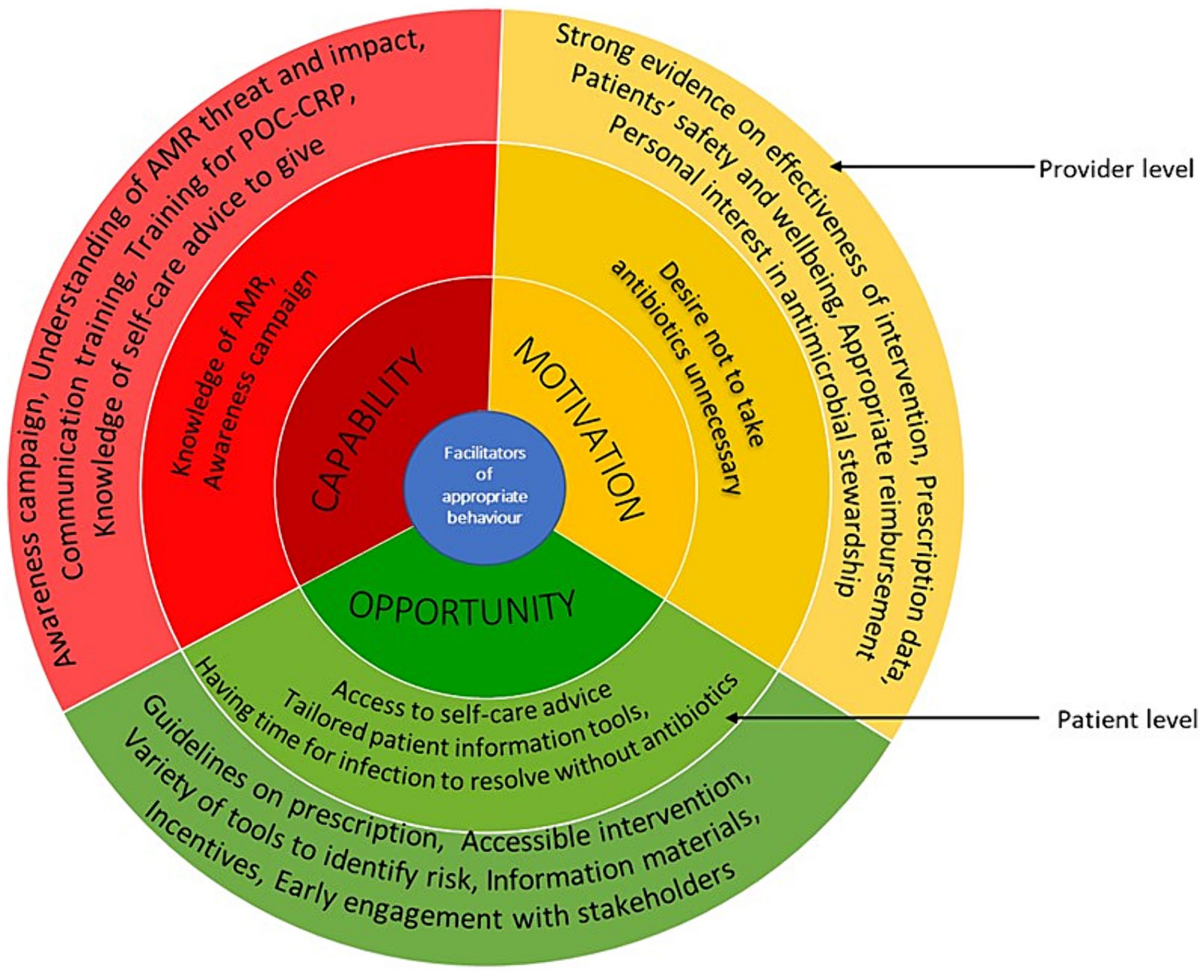
Facilitators of appropriate antimicrobial behaviour at healthcare professionals and patient levels mapped on to the COM-B model. The image summarises the facilitators identified from included studies. The outer layer refers to the provider level and inner layer refers to the patient level.

In the following section, we describe the barriers and facilitators based on the capability, opportunity, and motivation components of COM-B (see Figure 3 for barriers and Figure 4 for facilitators), starting with the provider level factors and then patient level factors. In Appendix 5, we present further details on the factors, including examples of types of interventions to mitigate the barriers based on the BCW.

## Provider level factors

### Capability

HCPs’ knowledge/understanding of AMR, antibiotics, threat and impact of AMR varied (23, 28, 29, 36, 43, 53, 77). A study among HCPs in paediatric emergency department in Canada reported that participants were unaware of their prescribing pattern and the scale and scope of the challenge of implementing antimicrobial stewardship in the emergency department (36). HCPs’ lack of awareness of the available interventions were also described (50, 53, 63, 72, 74, 76, 77). For example, many general practitioners in the United Kingdom were not aware of the Royal College of General Practitioners (RCGP) TARGET online courses and so they have not used them (53). Sometimes when they were aware of the available resources, they do not remember to use them, as reported across studies from United Kingdom and Australia (30, 40, 41). This is thought to be either due to busy routine or the fact that it was not part of their existing workflow (40, 41). Most HCPs had good understanding of C reactive protein (CRP) point of care testing, but some reported not knowing how to perform the test (40, 76). Some reported having the desire/ability to educate or persuade patients that no antibiotic is needed but sometimes were unable to do so and consequently prescribe antibiotics inappropriately (28, 73). There are issues with diagnostic uncertainty due to difficulty differentiating between viral infection and bacterial infection and recommendations based on the interventions do not always agree with their clinical judgement (23, 29, 43, 50, 51, 80).

Studies reported that communications trainings could help to increase clinicians’ confidence in not prescribing antibiotics (79), and improve general consulting style (43). Training HCPs to perform CRP point-of-care tests including refresher trainings were also reported two in United Kingdom studies (40, 51). General practitioners in United Kingdom and France highlighted that the knowledge of the public is an important issue and awareness campaigns should target both professionals and general public (45, 53, 67).

### Opportunity

Resources such as posters, printed decision aids, leaflets, booklets and videos were often used (23, 25, 27–30, 41, 43, 53, 72, 74, 76, 77, 79). Some clinicians used patient information sheets to reinforce their decision making/consultation discussion and provide self-help advice to patients (25, 27, 29, 30, 41, 43, 67, 77). However, there were concerns that sometimes the clinician’s treatment plan and the booklet messages differ which would create confusion (43). Computer based prompts and clinical prediction rules were highlighted to be particularly useful for less experienced staff who may not be very familiar with guidelines (50, 63). Many HCPs reported that resources such as clinical prediction rules and CRP point-of care testing helped them to manage patients’ expectation by providing evidence as to whether or not antibiotics are required, providing an objective measure to support judgement, reducing diagnostic uncertainties, supporting shared decision making and facilitating patient education around antibiotics (27, 29, 40, 44, 50, 51, 63, 73, 76). However, HCPs do not always use the tools. For example, some participants in studies from Germany and United Kingdom noted that their professional experience influences their decisions more than the guideline recommendation and clinical prediction rules (50, 73). Studies across United Kingdom and United States reported some participants feel that interventions, such as CRP testing, impact on the workflow and workload (23, 44, 51). Challenges of financing CRP point-of-care testing and the need for test cartridges to be refrigerated were also reported in three United Kingdom studies (40, 44, 51).

Lack of time was a major issue as HCPs have limited time with patients and utilising the interventions often adds to the time pressure (23, 27–29, 36, 40, 41, 44, 45, 50, 51, 53, 63, 72, 74, 76). Some clinicians in a study in Germany used delayed prescription due to diagnostic uncertainty or when the potential for follow-up visits was limited, such as planned vacations, public holidays (73). In another study in the United Kingdom, ‘rescue packs’ were provided to patients to manage acute exacerbations of chronic obstructive pulmonary disease at home (44). Perceived pressure from patients and other stakeholders (such as parents or carers) also contributed to inappropriate prescribing in studies across Australia, Canada and Germany (29, 36, 73). The need to support another HCP’s prescribing decision was also a barrier to appropriate behaviour (36). Studies from China and the United Kingdom reported lack of clarity regarding who will be responsible to take action and lack of monitoring of antimicrobial stewardship programmes (53, 80).

Improved accessibility of interventions is important (40, 44, 51, 63, 76). Simple, user-friendly, computer-based clinical decision support systems which are unintrusive and integrated into existing workflow were reported to be helpful (36, 50, 63). For printed materials, making them aesthetically appealing encouraged use (30). Clinicians in Australia reported that having a variety of tools so that they could choose what fits their communication style or patient preferences/needs was useful (29, 30).

### Motivation

Some HCPs believe that patients want antibiotics and will not be satisfied if they do not get them (23, 40, 43, 44, 51, 53, 73). Some studies highlighted the desire to satisfy patients due to the business nature of practises and fear of losing patients to other practises (29, 40). Some physicians in a study from Germany reported having emotional concerns and guilt when they do not administer a treatment or when they recommend non-prescription medicinal products which will cost patients money (73). Some physicians believe strategies such as delayed prescribing and rescue packs inappropriately shift responsibility of clinical decisions to patients and some patients find it difficult to understand when to use the antibiotics (44, 73). Also, patients may use the antibiotic immediately rather than wait (76). Some general practitioners in the United Kingdom are concerned that reducing antimicrobial prescribing would result in an increase in hospital admissions, so they prescribe antimicrobials to avoid missing infections or to avoid patient’s conditions worsening (44, 53). Studies from the United Kingdom and Australia reported some are fearful of litigation (29, 44).

Lack of confidence/trust/belief in the usefulness of an intervention or believing that an intervention provides no added value were also barriers to their use (23, 27, 29, 40, 63, 74). For example, some clinicians in the United Kingdom did not use prompts because they felt they did not need them since they were already working in line with the guidelines (63). Some HCPs in the United States believed that over prescription is not an issue in their site (23). HCPs’ perception of their own role in controlling antibiotic use, advising patients and performing tests were also important (28). In the case of electronic health record decision support systems, alert fatigue was a common issue as HCPs in a study from Canada reported that frequent pop-up alerts were disruptive to workflow, and the alerts are ignored (36).

In one study, it was suggested that showing HCPs data on their prescribing was potentially a useful strategy to motivate them to change practise (36). Another study highlighted that general practitioners would be more likely to use clinical prediction rules if there was strong evidence supporting its effectiveness and it has been adequately validated and tested in the primary care population (50). General practitioners in United Kingdom and Australia believe patients appreciate delayed prescribing as it provides them with a safety net and can prevent patients from getting worse (29, 44). In one study in France, HCPs (family physicians) requested to be paid for informing patients on why no antibiotics were being prescribed for them (45). However, in another study in the United Kingdom, HCPs (general practitioners) felt monetary incentives are not needed (50). Appropriate reimbursement for CRP point of care testing could be useful, although careful consideration is required since inadequate reimbursement systems may encourage inappropriate use or overuse (51). In a study that used antibiotic champions in the United Kingdom, it was reported that those who volunteered and had dedicated time for antimicrobial stewardship were more enthusiastic and engaged better with the intervention materials compared to those who were nominated (76).

## Patient level factors

### Capability

Knowledge about AMR, antibiotics and self-care among patients varied (25, 29, 30, 41, 43, 44, 67). For example, some patients in a United Kingdom study did not understand that AMR could be passed to others (67). In another United Kingdom study, patients felt the information provided in the materials were things they knew already and issues with receiving conflicting messages from clinicians were also reported (43). HCPs in Australia also noted that some patients may not have technology skills necessary to access electronic/online materials (30).

### Opportunity

Tools, such as posters, leaflets and decision aids, which are used by HCPs during consultation were reported to be useful in improving patient knowledge (25, 29, 30, 41, 43, 67). However, some may view the booklet as a way to discourage them from seeing the doctor as reported in a United Kingdom study (43). Clinicians in the United Kingdom reported that CRP is a way of educating patients for the future and gave patients confidence (40, 44). A study in Australia reported some childcare centre regulations allow children with certain symptoms return to the setting if they are on antibiotics, this was thought to be one of the reasons parents often demand antibiotics (29). When information was provided on tablets in waiting areas, patients in Germany were concerned about risk of infection (72, 74).

Access to self-care advice, pharmacy, facilities to self-care at home, information on self-care and when to get help and having the time for respiratory tract infections to get better on their own are necessary for appropriate antibiotic behaviour (41). Patients suggested having information sheets, posters and booklets in the general practise waiting rooms and pharmacies would be useful (30).

### Motivation

Some patients believe in the issue of AMR, the consequences and side effects (41). Studies from the United Kingdom and United States reported that some patients do consult with a prior determination that they need antibiotics and were disappointed when they did not receive a prescription, especially when they felt they did not receive a thorough examination or sufficient information (42, 43). However, a study reported that parents desire thorough examination and reassurance rather than specific treatment when their children were unwell (43). Patient’s trust in the HCPs seems to encourage them to follow the professional’s advice as reported in a United Kingdom study (67). Another United Kingdom study reported that many patients do not want to take antibiotics unnecessarily (44). A study in Germany reported some patients may find it difficult to stand up against HCPs’ suggestion even if they feel it is wrong (74).

Overall, issues hindering appropriate behaviours for both HCPs and patients are wide-ranging. Based on the BCW, a broad range of intervention types can be applied (12). For example, education, training, environmental restructuring (such as, using prompts), restriction (using rules and regulations to reduce inappropriate prescription), enablement (such as audit and feedback on prescribing behaviour), modelling/champions and incentivisation (12). A list of the intervention types that could be used to mitigate the issues identified and for each COM-B components are presented in Appendix 5.

## Discussion

This review consolidates existing evidence on the interventions supporting HCPs in their interaction with patients/public, employing a theoretical framework to group the barriers and facilitators of appropriate behaviour. We identified various interventions. Despite the availability of interventions, our findings show factors that impede or enhance the ability of both HCPs and patients to utilise/benefit from the interventions and make informed decisions about antimicrobial use. We grouped these barriers and facilitators into capability, opportunity, and motivation, providing a foundation for future work to tackle AMR.

One of the most frequently reported issues relating to capability is both HCPs and patient’s knowledge/awareness and understanding of AMR, antimicrobials and the impact of AMR, which varied across studies with no clear pattern (23, 28, 29, 36, 43, 53, 77). This suggests the need for strategies to improve knowledge among both HCPs and patients (45, 53, 67). Studies have shown that current AMR campaigns, including World Antimicrobial Awareness Week, do not result in significant public awareness or behaviour change (82, 83). Despite understanding the importance of not prescribing antibiotics unnecessarily, some HCPs reported difficulty persuading patients leading to inappropriate prescribing (28, 73). Several studies indicated that training could enhance clinicians’ ability to avoid inappropriate prescriptions and improve consulting styles (40, 43, 79). There are various resources available to support but lack of awareness of available resources or forgetting about them is reported in several studies (50, 53, 63, 72, 74, 76, 77). This underscores the need for immediate action from healthcare leaders and policymakers to devise strategies addressing these challenges that impact on capability. These strategies should extend beyond education or training initiatives and incorporate measures to ensure the sustained implementation of any positive changes.

In terms of opportunity, time constraints is a frequent issue among HCPs (23, 27–29, 36, 40, 41, 44, 45, 50, 51, 53, 63, 72, 74, 76). As shown in the findings, various resources such as posters, decision aids, and leaflets were available, and patients reported their potential usefulness (30). However, HCPs are often under pressure to manage consultations efficiently and in many contexts, time is often strictly restricted. This may hinder their ability to thoroughly assess the necessity of antimicrobials or to effectively communicate to patients why antimicrobials are unnecessary. Some HCPs are able to effectively use patient information sheets to reinforce discussions and provide self-help advice to patients (25, 27, 29, 30, 41, 43, 67, 77). Care must be taken to avoid inconsistencies between clinician’s treatment plans and messages in the leaflets (43). Improved accessibility of interventions, including providing simple, visually appealing materials is important as these aspects were considered beneficial (40, 44, 51, 63, 76). Having a variety of tools to accommodate different communication styles or patient preferences/needs was considered useful (29, 30). Research highlighted uncertainties regarding who will be responsible to take action and the absence of effective monitoring of antimicrobial stewardship programmes (53, 80). Clarifying the roles of individuals and the role of various organisations, in tackling AMR would be helpful (84). In one study in Australia, it was reported that some childcare centre regulations allow children with certain symptoms return to the setting if they are on antibiotics, this may drive parents to desire antibiotics (29). This is also true in the United Kingdom (85). This exemplifies the need to review policies and factors that may impact on antimicrobial use across various sectors.

Regarding motivation, the perception that patients want antibiotics is a common issue which spans across various contexts (23, 40, 43, 44, 51, 53, 73). For example, in some instances where HCPs and patients have good knowledge of AMR and use available interventions HCPs frequently assume patients expect antibiotics and feel pressure to prescribe antibiotics even when they are not clinically indicated (23, 40, 43, 44, 51, 53, 73). Whereas patients do not always want antibiotics, sometimes they only want reassurance (43). The overestimation of patients’ desire for antibiotics have also been highlighted by others (86). HCPs have also reported fear of patient’s condition deteriorating and fear of litigation as a reason for prescribing antibiotics, even when they would have preferred not to (87). General practitioners viewed delayed prescribing favourably, as it offers a safety net (29, 44). Monetary incentives were suggested, however, opinions varied regarding the necessity for monetary incentives for behaviour change among HCPs (45, 50, 51). Careful considerations must be paid to incentives across different sectors to prevent propagation of inappropriate behaviours. For instance, while the health and governmental domains aim to encourage the responsible use of antibiotics, pharmaceutical companies may have incentives aligned with increased usage (88).

Overall, navigating issues related to appropriate antimicrobial behaviour is a multifaceted challenge. As a result, a multifaceted approach is necessary to tackle all the components of behaviour drivers simultaneously to make meaningful improvement in antimicrobial prescribing behaviour, as with other interventions to change behaviour. Future studies should focus on the development of suitable strategies to improve the identified behaviour drivers among HCPs and the public, while also maximising the utilisation of existing interventions. Policymakers should encourage multidisciplinary collaboration among HCPs, patients, caregivers, and various organisational sectors to address the complexities of antimicrobial stewardship. This collaborative approach can facilitate the development and implementation of effective interventions to tackle AMR. It is worth noting that tackling AMR requires a global effort but there are currently inconsistencies regarding how antimicrobial prescriptions are regulated and enforced globally (4). It is important that health organisations and policy makers globally focus on developing appropriate interventions to improve psychological (individual motivations to act), social (collective support) and structural (capability and opportunity) conditions to achieve a continuous positive change (89).

We used a preregistered protocol and performed comprehensive searches of electronic databases and grey literature to minimise the risk of publication bias. The full-text screening phase of the study selection was performed in duplicate. Data extraction was checked by a second reviewer to ensure accuracy. We also used a theoretical framework to analyse the barriers and facilitators which facilitates the identification of possible intervention types which may be used to mitigate barriers identified. These are specific strengths of this review. However, since we limited to articles published in English Language due to limited time and resources, some potentially relevant studies which are not published in English Language may have been missed. Also, a single reviewer performed title and abstract screening, although 10% were double screened, we acknowledge that some potentially relevent studies may have inadvertently been overlooked. We conducted a quality assessment of the included articles to offer an overview of their overall methodological quality. However, we acknowledge that most of the quality criteria included in the quality assessment tools that we used, particularly for randomised controlled trials, focus on effectiveness which is not the focus of this review. Furthermore, although we are interested in interventions that aim to improve HCPs’ interaction with patients, many of the interventions in included studies had several components and the findings relating to the patient interaction components are not always presented differently. Therefore, most of the issues highlighted may not necessarily pertain to the patient interaction alone. We aimed to provide a comprehensive overview of the barriers and facilitators related to the utilisation of the interventions of interest and appropriate antimicrobial use among HCPs and patients. It is important to consider contextual factors when applying the review findings. Differences in interventions, and study populations prevent us from making exhaustive comparisons across countries. We categorised barriers and facilitators under capability, opportunity, or motivation but we are aware that some of the issues identified may cut across different components. We used COM-B framework to group the barriers and facilitators and highlighted potential intervention types which may be used to target the barriers.

Future work is needed to conduct an in-depth behavioural analysis to understand the behavioural drivers, use evidence-based approaches to prioritise the key issues to be addressed, examine how existing interventions tackle these issues, and identify opportunities for improvement. This may have to focus on individual context, as demonstrated in a previous systematic review (17). Such efforts will serve as a foundation for developing targeted interventions or improving existing ones in collaboration with relevant stakeholders to enhance HCPs and patient interaction to encourage appropriate behaviour.

This review identified a range of interventions that support HCPs to improve their interaction with patients in order to promote appropriate antimicrobial use. The barriers and facilitators identified covered all components of the COM-B model, providing a wide range of avenues for improvement. These findings should be considered when developing, implementing, or improving interventions to support HCPs in interacting with patients to promote appropriate antimicrobial behaviour.

## Data availability statement

The original contributions presented in the study are included in the article/Supplementary material, further inquiries can be directed to the corresponding author.

## Author contributions

AA: Data curation, Formal analysis, Funding acquisition, Investigation, Methodology, Project administration, Resources, Validation, Writing – original draft, Writing – review & editing. IG: Data curation, Formal analysis, Funding acquisition, Methodology, Writing – review & editing. JS: Data curation, Writing – review & editing. VA: Data curation, Writing – review & editing. AB: Writing – review & editing. MJ: Writing – review & editing. EG: Methodology, Writing – review & editing. DT: Methodology, Writing – review & editing. DA-O: Methodology, Writing – review & editing.
