# Supplementary material for: Improving healthcare professionals’ interactions with patients to tackle antimicrobial resistance: a systematic review of interventions, barriers, and facilitators
Source: Front Public Health. 2024 May 22;12:1359790. doi: 10.3389/fpubh.2024.1359790 (PMC11150712; doi:10.3389/fpubh.2024.1359790)
Supplement: Supplementary file 1 [file Table_1.DOCX]

Improving healthcare professionals’ interactions with patients to tackle antimicrobial resistance: a systematic review of interventions, barriers, and facilitators.

Abimbola Ayorinde, Iman Ghosh, Junaid Shaikh, Victoria Adetunji, Anna Brown, Mary Jordan, Ellie Gilham, Daniel Todkill, Diane Ashiru-Oredope

**Table of Contents**

[Appendix 1: Search Strategies 2](#_Toc140099519)

[MEDLINE (via Ovid) 2](#_Toc140099520)

[Embase (via Ovid) 2](#_Toc140099521)

[Science Citation Index and Social Sciences Citation Index (via Web of Science) 3](#_Toc140099522)

[Google Scholar (via Harzing’s Publish or Perish) 4](#_Toc140099523)

[Appendix 2: Excluded studies 5](#_Toc140099524)

[Appendix 3: Quality Assessment (Using Mixed Methods Appraisal Tool) 12](#_Toc140099525)

[Appendix 4: Interventions 15](#_Toc140099526)

[Appendix 5: Barriers, facilitators, and corresponding intervention types 32](#_Toc140099527)

[References 37](#_Toc140099528)

# Appendix 1: Search Strategies

## MEDLINE (via Ovid)

Date searched: 31/01/23

Ovid MEDLINE(R) ALL <1946 to January 30, 2023>

1 exp Anti-Infective Agents/ 1795712

2 (antibiotic* or anti biotic* or antibacterial* or anti bacterial* or antimicrobial* or anti microbial*).kf,tw. 631028

3 1 or 2 2061557

4 Inappropriate Prescribing/ or Practice Patterns, Physicians'/ 69848

5 Antimicrobial Stewardship/ 3221

6 Drug Resistance, Microbial/ or exp Drug Resistance, Bacterial/ 159580

7 ((antibiotic* or anti biotic* or antibacterial* or anti bacterial* or antimicrobial* or anti microbial*) adj3 ("use" or prescri* or overuse or overprescri* or usage or consum* or dispens*)).kf,tw. 63993

8 ((antibiotic* or anti biotic* or antibacterial* or anti bacterial* or antimicrobial* or anti microbial*) adj1 (resistan* or stewardship)).kf,tw. 96473

9 4 or 5 or 6 or 7 or 8 320765

10 3 and 9 [ antimicrobial use/prescribing] 237975

11 exp Health Personnel/ or Practice Patterns, Physicians'/ 655960

12 (doctor? or medic? or physician* or pharmacist* or prescriber* or general practitioner* or GP or GPs or clinician*).kf,tw. 976852

13 ((Health* or medical* or clinical) adj2 (professional* or practitioner* or personnel or staff or worker?)).kf,tw. 275936

14 ((hospital? or "primary care" or "general practice?" or "ambulatory care" or "secondary care" or "acute care") and (personnel or staff or employee?)).kf,tw. 89978

15 11 or 12 or 13 or 14 [HCPs] 1650788

16 ((antimicrobial resistance or antibacterial resistance or AMR or anti microbial resistance or anti bacterial resistance or antibiotic resistance or anti biotic resistance or antibiotic prescribing or anti biotic prescribing or antibacterial prescribing or anti bacterial prescribing) adj4 (intervention* or program* or initiative*)).kf,tw. 1214

17 (stewardship adj3 (intervention* or program* or initiative*)).kf,tw. 4069

18 (decision aid* or decision support or checklist? or clinical score? or clinical scoring or reminder? or (clinical adj2 rule?) or toolkit?).kf,tw. 117738

19 Decision Support Systems, Clinical/ or exp Decision Support Techniques/ or Checklist/ or Clinical Protocols/ or Reminder Systems/ or Decision Making, Shared/ 132948

20 Education, Medical, Continuing/ or exp Inservice Training/ 54900

21 ((communicati* or consultation or consulting or practice based) adj2 (training or workshop? or seminar? or webinar?)).kf,tw. 4820

22 shared decision making.kf,tw. 13312

23 (barrier* adj2 (facilitator* or enabler*)).kf,tw. 17175

24 behavio?r change.kf,tw. 20572

25 16 or 17 or 18 or 19 or 20 or 21 or 22 or 23 or 24 [interventions aimed at HCPs interactions with patients, barriers/facilitators] 337643

26 10 and 15 and 25 3083

27 limit 26 to (english language and yr="2010 -Current") 2562

Note: Lines 1-10 are based on search terms used in Borek AJ, Wanat M, Atkins L, Sallis A, Ashiru-Oredope D, Beech E*, et al.* Optimising antimicrobial stewardship interventions in English primary care: a behavioural analysis of qualitative and intervention studies. *BMJ Open* 2020;**10**(12):e039284. <http://dx.doi.org/10.1136/bmjopen-2020-039284>

## Embase (via Ovid)

Date searched: 31/01/23

Embase <1974 to 2023 January 30>

1 *antiinfective agent/ or *antibiotic agent/ 183529

2 (antibiotic* or anti biotic* or antibacterial* or anti bacterial* or antimicrobial* or anti microbial*).kf,tw. 813869

3 1 or 2 859167

4 exp unnecessary prescribing/ 394

5 *antimicrobial stewardship/ 4430

6 exp antibiotic resistance/ 202551

7 ((antibiotic* or anti biotic* or antibacterial* or anti bacterial* or antimicrobial* or anti microbial*) adj3 ("use" or prescri* or overuse or overprescri* or usage or consum* or dispens*)).kf,tw. 85819

8 ((antibiotic* or anti biotic* or antibacterial* or anti bacterial* or antimicrobial* or anti microbial*) adj1 (resistan* or stewardship)).kf,tw. 120790

9 4 or 5 or 6 or 7 or 8 299438

10 3 and 9 231833

11 health care personnel/ or exp medical personnel/ or clinical practice/ or exp pharmacist/ 1685875

12 (doctor? or medic? or physician* or pharmacist* or prescriber* or general practitioner* or GP or GPs or clinician*).kf,tw. 1411577

13 ((Health* or medical* or clinical) adj2 (professional* or practitioner* or personnel or staff or worker?)).kf,tw. 358158

14 ((hospital? or "primary care" or "general practice?" or "ambulatory care" or "secondary care" or "acute care") and (personnel or staff or employee?)).kf,tw. 134616

15 11 or 12 or 13 or 14 2776610

16 ((antimicrobial resistance or antibacterial resistance or AMR or anti microbial resistance or anti bacterial resistance or antibiotic resistance or anti biotic resistance or antibiotic prescribing or anti biotic prescribing or antibacterial prescribing or anti bacterial prescribing) adj4 (intervention* or program* or initiative*)).kf,tw. 1638

17 (stewardship adj3 (intervention* or program* or initiative*)).kf,tw. 6442

18 (decision aid* or decision support or checklist? or clinical score? or clinical scoring or reminder? or (clinical adj2 rule?) or toolkit?).kf,tw. 162809

19 decision support system/ or reminder system/ or shared decision making/ 42362

20 ((communicati* or consultation or consulting or practice based) adj2 (training or workshop? or seminar? or webinar?)).kf,tw. 6564

21 shared decision making.kf,tw. 18733

22 (barrier* adj2 (facilitator* or enabler*)).kf,tw. 21532

23 behavio?r change.kf,tw. 24664

24 16 or 17 or 18 or 19 or 20 or 21 or 22 or 23 249999

25 10 and 15 and 24 4436

26 limit 25 to (english language and yr="2010 -Current") 4069

## Science Citation Index and Social Sciences Citation Index (via Web of Science)

Date searched: 31/01/23

Web of Science Core Collection: SCI-EXPANDED , SSCI

((TS=((antibiotic* OR "anti biotic*" OR antibacterial* OR "anti bacterial*" OR antimicrobial* OR "anti microbial*" ) NEAR/2 (use OR prescri* OR overuse OR overprescri* OR usage OR consum* OR dispens* ))) OR (TS=((antibiotic* OR "anti biotic*" OR antibacterial* OR "anti bacterial*" OR antimicrobial* OR "anti microbial*" ) NEAR/0 (resistan* OR stewardship )))) AND ((TS=(doctor$ OR medic$ OR physician* OR pharmacist* OR prescriber* OR "general practitioner*" OR GP OR GPs OR clinician* )) OR (TS=((Health* OR medical* OR clinical ) NEAR/1 (professional* OR practitioner* OR personnel OR staff OR worker$ ))) OR (TS=((hospital$ OR "primary care" OR "general practice$" OR "ambulatory care" OR "secondary care" OR "acute care" ) AND (personnel OR staff OR employee$ )))) AND ((TS=(("antimicrobial resistance" OR "antibacterial resistance" OR AMR OR "anti microbial resistance" OR "anti bacterial resistance" OR "antibiotic resistance" OR "anti biotic resistance" OR "antibiotic prescribing" OR "anti biotic prescribing" OR "antibacterial prescribing" OR "anti bacterial prescribing" ) NEAR/3 (intervention* OR program* OR initiative* ))) OR (TS=(stewardship NEAR/2 (intervention* OR program* OR initiative* ))) OR (TS=("decision aid*" OR "decision support" OR checklist$ OR "clinical score$" OR "clinical scoring" OR reminder$ OR (clinical NEAR/1 rule$ ) OR toolkit$ )) OR (TS=((communicati* OR consultation OR consulting OR "practice based" ) NEAR/1 (training OR workshop$ OR seminar$ OR webinar$ ))) OR (TS="shared decision making") OR (TS=(barrier* NEAR/1 (facilitator* OR enabler* ))) OR (TS="behavio$r change")) and 2023 or 2022 or 2021 or 2020 or 2019 or 2018 or 2017 or 2016 or 2015 or 2014 or 2013 or 2012 or 2011 or 2010 (Publication Years) and English (Languages)

1,862 results

Note: The Ovid Medline search strategy was translated for use in the Web of Science interface with the aid of the Polyglot Search Translator: Clark JM, Sanders S, Carter M, Honeyman D, Cleo G, Auld Y, et al. Improving the translation of search strategies using the Polyglot Search Translator: a randomized controlled trial. J Med Libr Assoc 2020;108(2):195-207. <http://dx.doi.org/10.5195/jmla.2020.834>

## Google Scholar (via Harzing’s Publish or Perish)

Searched using Harzing’s Publish or Perish (Windows GUI edition) version 8: Harzing AW. Publish or Perish 8. 2021. Available from: <https://harzing.com/resources/publish-or-perish>

Date searched: 01/02/23

3 separate searches were run, with the maximum number of results for each selected based on test searches in the Google Scholar interface (<https://scholar.google.co.uk/>)

*Search 1:*

antimicrobial|antibacterial|antibiotic resistance|stewardship|prescribing intervention|program|programme|initiative professionals|doctors|medics|clinicians|pharmacists|prescribers|physicians|practitioners|staff|personnel|employees|workers

Years 2010-2023

Maximum number of results: 300

*Search 2:*

antimicrobial|antibacterial|antibiotic resistance|stewardship|prescribing "decision support"|"shared decision making"|"barriers and facilitators"

Years 2010-2023

Maximum number of results: 200

*Search 3:*

antimicrobial|antibacterial|antibiotic resistance|stewardship|prescribing checklist|toolkit

Years 2010-2023

Maximum number of results: 100

Endnote 20’s ‘Find reference updates’ function was used to retrieve further details of each record.

# Appendix 2: Excluded studies

| **Title** | **Authors** | **Year** | **Reasons for exclusion** |
| --- | --- | --- | --- |
| Antimicrobial Stewardship Programs (ASPS) in pediatric primary care | Albanil Ballesteros, M. R. and Rodriguez Arranz, C. and Jimenez Ales, R. and Ares Alvarez, J. and Munoz Hiraldo, E. and Lupiani Castellanos, P. and Martinez Chamorro, M. J. and Garcia Vera, C. and Suarez Arrabal, M. C. | 2023 | Foreign Language |
| Physicians with access to point-of-care tests significantly reduce the antibiotic prescription for common cold | Llor, C. and Hern and ez, S. and Cots, J. M. and Bjerrum, L. and Gonzalez, B. and Garcia, G. and Alcantara, J. D. and Guerra, G. and Cid, M. and Gomez, M. and Ortega, J. and Perez, C. and Arranz, J. and Monedero, M. J. and Paredes, J. and Pineda, V. | 2013 | Foreign Language |
| A Mixed Methods Approach to Tailoring Evidence-Based Guidance for Antibiotic Stewardship to One Medical System | Patterson, E. S. and Dewart, C. M. and Stevenson, K. and Mbodj, A. and Lustberg, M. and Hade, E. M. and Hebert, C. | 2018 | No relevant intervention |
| A mixed methods study of the barriers and enablers in implementing antimicrobial stewardship programmes in Australian regional and rural hospitals | James, R. and Luu, S. and Avent, M. and Marshall, C. and Thursky, K. and Buising, K. | 2015 | No relevant intervention |
| A National Survey of Critical Care Physicians' Knowledge, Attitudes, and Perceptions of Antimicrobial Stewardship Programs | Steinberg, M. and Dresser, L. D. and Daneman, N. and Smith, O. M. and Matte, A. and Marinoff, N. and Bell, C. M. and Morris, A. M. | 2016 | No relevant intervention |
| A national survey of Irish community pharmacists' antimicrobial stewardship views and activity | Barbosa, T. M. and O'Regan, U. and Fleming, A. | 2020 | No relevant intervention |
| A process evaluation of the UK-wide Antibiotic Guardian campaign: developing engagement on antimicrobial resistance | Bhattacharya, A. and Hopkins, S. and Sallis, A. and Budd, E. L. and Ashiru-Oredope, D. | 2017 | No relevant intervention |
| A questionnaire based survey among pharmacy practitioners to evaluate the level of knowledge and confidence towards antimicrobial stewardship | Gillani, S. W. and Shahwan, M. K. S. and Szollosi, D. E. | 2022 | No relevant intervention |
| A Seat at the Table: Delivering Effective Infectious Diseases and Antimicrobial Stewardship Education to Advanced Practice Providers at an Academic Medical Center | Lee, M. S. L. and Stead, W. | 2022 | No relevant intervention |
| Accelerating reductions in antimicrobial resistance: Evaluating the effectiveness of an intervention program implemented by an infectious disease consultant | Kishida, N. and Nishiura, H. | 2020 | No relevant intervention |
| Addressing antimicrobial resistance in Nigerian hospitals: exploring physicians prescribing behavior, knowledge, and perception of antimicrobial resistance and stewardship programs | Babatola, A. O. and Fadare, J. O. and Olatunya, O. S. and Obiako, R. and Enwere, O. and Kalungia, A. and Ojo, T. O. and Sunmonu, T. A. and Desalu, O. and Godman, B. | 2021 | No relevant intervention |
| An antibiotic stewardship program in a French teaching hospital | Mondain, V. and Lieutier, F. and Dumas, S. and Gaudart, A. and Fosse, T. and Roger, P. M. and Bernard, E. and Farhad, R. and Pulcini, C. | 2013 | No relevant intervention |
| Antibiotic stewardship based on education: minor impact on knowledge, perception and attitude | Kjaersgaard, M. and Leth, R. A. and Udupi, A. and Ank, N. | 2019 | No relevant intervention |
| Antibiotic stewardship program in Pakistan: a multicenter qualitative study exploring medical doctors' knowledge, perception and practices | Atif, M. and Ihsan, B. and Malik, I. and Ahmad, N. and Saleem, Z. and Sehar, A. and Babar, Z. U. | 2021 | No relevant intervention |
| Antimicrobial stewardship across 47 South African hospitals: an implementation study | Brink, A. J. and Messina, A. P. and Feldman, C. and Richards, G. A. and Becker, P. J. and Goff, D. A. and Bauer, K. A. and Nathwani, D. and van den Bergh, D. and Netcare Antimicrobial Stewardship Study, Alliance | 2016 | No relevant intervention |
| Antimicrobial stewardship in the treatment of skin and soft tissue infections | Gibbons, J. A. and Smith, H. L. and Kumar, S. C. and Duggins, K. J. and Bushman, A. M. and Danielson, J. M. and Yost, W. J. and Wadle, J. J. | 2017 | No relevant intervention |
| Antimicrobial Stewardship Initiatives Throughout Europe: Proven Value for Money | Oberje, E. J. M. and Tanke, M. A. C. and Jeurissen, P. P. T. | 2017 | No relevant intervention |
| Antimicrobial stewardship program: An adventure from erratic towards utopia | Mohammed, Z. A. and Eshwara, V. K. and Varma, M. and Sekhar, S. and Mukhopadhyay, C. | 2018 | No relevant intervention |
| Antimicrobial stewardship programs in community hospitals: The evidence base and case studies | Ohl, C. A. and Dodds Ashley, E. S. | 2011 | No relevant intervention |
| Antimicrobial stewardship solutions with a smart innovative tool | Shawki, M. A. and AlSetohy, W. M. and Ali, K. A. and Ibrahim, M. R. and El-Husseiny, N. and Sabry, N. A. | 2021 | No relevant intervention |
| Association between Physicians' Perception of Shared Decision Making with Antibiotic Prescribing Behavior in Primary Care in Hubei, China: A Cross-Sectional Study | Wang, D. and Liu, C. and Wang, X. and Zhang, X. | 2020 | No relevant intervention |
| Automated alerts coupled with antimicrobial stewardship intervention lead to decreases in length of stay in patients with gram-negative bacteremia | Pogue, J. M. and Mynatt, R. P. and Marchaim, D. and Zhao, J. J. and Barr, V. O. and Moshos, J. and Sunkara, B. and Chopra, T. and Chidurala, S. and Kaye, K. S. | 2014 | No relevant intervention |
| Behavioral Economics Interventions to Improve Outpatient Antibiotic Prescribing for Acute Respiratory Infections: a Cost-Effectiveness Analysis | Gong, C. L. and Zangwill, K. M. and Hay, J. W. and Meeker, D. and Doctor, J. N. | 2019 | No relevant intervention |
| Changes in antimicrobial prescribing behavior after the introduction of the antimicrobial stewardship program: A pre- and post-intervention survey | Chavada, R. and Walker, H. N. and Tong, D. and Murray, A. | 2017 | No relevant intervention |
| Clinical benefit of infectious diseases consultation: a monocentric prospective cohort study | de La Blanchardiere, A. and Boutemy, J. and Thibon, P. and Michon, J. and Verdon, R. and Cattoir, V. | 2012 | No relevant intervention |
| Critical points in the pathway of antibiotic prescribing in a children's hospital: the Antibiotic Mapping of Prescribing (ABMAP) study | Bashir, A. and Gray, J. and Bashir, S. and Ahmed, R. and Theodosiou, E. | 2019 | No relevant intervention |
| Design and analysis of a pharmacist-enhanced antimicrobial stewardship program in Thailand | Apisarnthanarak, A. and Lapcharoen, P. and Vanichkul, P. and Srisaeng-Ngoen, T. and Mundy, L. M. | 2015 | No relevant intervention |
| Discrepancies between qualitative and quantitative evaluation of randomised controlled trial results: achieving clarity through mixed methods triangulation | Tonkin-Crine, S. and Anthierens, S. and Hood, K. and Yardley, L. and Cals, J. W. and Francis, N. A. and Coenen, S. and van der Velden, A. W. and Godycki-Cwirko, M. and Llor, C. and Butler, C. C. and Verheij, T. J. and Goossens, H. and Little, P. and consortium, Grace Intro Champ | 2016 | No relevant intervention |
| Evaluating the Impact of a Pharmacist-Led Antimicrobial Stewardship Intervention at Discharge in a Community, Nonteaching Hospital | Manis, M. M. and Kyle, J. A. and Dajani, D. and Pan, K. and Hughes, P. J. and Adunlin, G. and Allen, L. N. and Leonard, C. E. | 2022 | No relevant intervention |
| Feasibility and effectiveness of a low cost campaign on antibiotic prescribing in Italy: community level, controlled, non-randomised trial | Formoso, G. and Paltrinieri, B. and Marata, A. M. and Gagliotti, C. and Pan, A. and Moro, M. L. and Capelli, O. and Magrini, N. and Group, Locaal Study | 2013 | No relevant intervention |
| Feasibility cluster randomised controlled trial of a within-consultation intervention to reduce antibiotic prescribing for children presenting to primary care with acute respiratory tract infection and cough | Blair, P. S. and Turnbull, S. and Ingram, J. and Redmond, N. and Lucas, P. J. and Cabral, C. and Hollinghurst, S. and Dixon, P. and Peters, T. and Horwood, J. and Little, P. and Francis, N. A. and Gilbertson, A. and Jameson, C. and Hay, A. D. | 2017 | No relevant intervention |
| Feasibility of a Nursing Home Antibiotic Stewardship Intervention | Baier, R. R. and Jump, R. L. P. and Zhang, T. and Kabbani, S. and Gifford, D. R. and Gravenstein, S. | 2022 | No relevant intervention |
| Impact of Antibiotic Time-Outs in Multidisciplinary ICU Rounds for Antimicrobial Stewardship Program on Patient Survival: A Controlled Before-and-After Study | Mishima, Y. and Nawa, N. and Asada, M. and Nagashima, M. and Aiso, Y. and Nukui, Y. and Fujiwara, T. and Shigemitsu, H. | 2023 | No relevant intervention |
| Impact of targeted educational intervention towards public knowledge and perception of antibiotic use and resistance in the state of Perak, Malaysia | Thong, K. S. and Chang, C. T. and Lee, M. and Lee, J. C. Y. and Tan, H. S. and Shafie, A. A. | 2021 | No relevant intervention |
| Knowledge and perceptions of antimicrobial resistance and antimicrobial stewardship among staff at a national cancer referral center in Uganda | Gulleen, E. A. and Lubwama, M. and Komakech, A. and Krantz, E. M. and Liu, C. and Phipps, W. | 2022 | No relevant intervention |
| Knowledge, attitude and practices of clinicians, nurses and pharmacists regarding antimicrobial stewardship: A five center survey from India | Mane, A. and Kamat, S. and Thanusubramanian, H. | 2021 | No relevant intervention |
| Outcomes of multisite antimicrobial stewardship programme implementation with a shared clinical decision support system | Bond, S. E. and Chubaty, A. J. and Adhikari, S. and Miyakis, S. and Boutlis, C. S. and Yeo, W. W. and Batterham, M. J. and Dickson, C. and McMullan, B. J. and Mostaghim, M. and Li-Yan Hui, S. and Clezy, K. R. and Konecny, P. | 2017 | No relevant intervention |
| Outcomes of the PIRASOA programme, an antimicrobial stewardship programme implemented in hospitals of the Public Health System of Andalusia, Spain: an ecologic study of time-trend analysis | Rodriguez-Bano, J. and Perez-Moreno, M. A. and Penalva, G. and Garnacho-Montero, J. and Pinto, C. and Salcedo, I. and Fern and ez-Urrusuno, R. and Neth, O. and Gil-Navarro, M. V. and Perez-Milena, A. and Sierra, R. and Estella, A. and Lupion, C. and Irastorza, A. and Marquez, J. L. and Pascual, A. and Rojo-Martin, M. D. and Perez-Lozano, M. J. and Valencia-Martin, R. and Cisneros, J. M. and Group, Pirasoa Programme | 2020 | No relevant intervention |
| Patient and physician attitudes regarding risk and benefit in streamlined development programmes for antibacterial drugs: a qualitative analysis | Holl and , T. L. and Mikita, S. and Bloom, D. and Roberts, J. and McCall, J. and Collyar, D. and Santiago, J. and Tiernan, R. and Toerner, J. | 2016 | No relevant intervention |
| Patient engagement with infection management in secondary care: a qualitative investigation of current experiences | Rawson, T. M. and Moore, L. S. and Hern and ez, B. and Castro-Sanchez, E. and Charani, E. and Georgiou, P. and Ahmad, R. and Holmes, A. H. | 2016 | No relevant intervention |
| Pediatric Urgent Care Providers' Approach to Antibiotic Stewardship: A National Survey | Hamdy, R. F. and Nedved, A. and Fung, M. and Fleming-Dutra, K. E. and Liu, C. M. and Obremskey, J. and Montalbano, A. | 2022 | No relevant intervention |
| Perceptions of Antibiotic Therapy Among Nursing Home Residents: Perspectives of Caregivers and Residents in a Mixed Exploratory Study | Ahouah, M. and Lartigue, C. and Rothan-Tondeur, M. | 2019 | No relevant intervention |
| Physicians' attitudes on the implementation of an antimicrobial stewardship program in Lebanese hospitals | Sayegh, N. and Hallit, S. and Hallit, R. and Saleh, N. and Zeidan, R. K. | 2021 | No relevant intervention |
| Reducing antibiotic for child upper respiratory infections in rural china: An RCT, process evaluation and cost-effectiveness analysis | Wei, X. and Zhang, Z. and Walley, J. and Hicks, J. and Dawkins, B. and Zeng, J. and Lin, M. | 2018 | No relevant intervention |
| Self-Assessment of Antimicrobial Stewardship in Primary Care: Self-Reported Practice Using the TARGET Primary Care Self-Assessment Tool | Owens, R. and Jones, L. F. and Moore, M. and Pilat, D. and McNulty, C. | 2017 | No relevant intervention |
| Self-medicated broad spectrum antibiotics in rural communities in kano-nigeria: A cross-sectional survey | Yusuf, I. and Jobbi, Y. D. and Arzai, A. H. and Shuaib, M. and Ahmad, A. S. | 2019 | No relevant intervention |
| Self-Reported Antimicrobial Stewardship Practices in Primary Care Using the TARGET Antibiotics Self-Assessment Tool | Jones, L. F. and Verl and er, N. Q. and Lecky, D. M. and Altaf, S. and Pilat, D. and McNulty, C. | 2020 | No relevant intervention |
| Snapshot of antimicrobial stewardship in Australian hospitals | Chen, A. W. J. and Khumra, S. and Eaton, V. and Kong, D. C. M. | 2010 | No relevant intervention |
| Social and Contextual Influences on Antibiotic Prescribing and Antimicrobial Stewardship: A Qualitative Study with Clinical Commissioning Group and General Practice Professionals | Borek, A. J. and Anthierens, S. and Allison, R. and McNulty, C. A. M. and Anyanwu, P. E. and Costelloe, C. and Walker, A. S. and Butler, C. C. and Tonkin-Crine, S. and On Behalf Of The Step-Up Study, Team | 2020 | No relevant intervention |
| Social media as a tool for antimicrobial stewardship | Pisano, J. and Pettit, N. and Bartlett, A. and Bhagat, P. and Han, Z. and Liao, C. and L and on, E. | 2016 | No relevant intervention |
| Strategies for improving antibiotic use in Qatar: a survey of pharmacists' perceptions and experiences | Pawluk, S. and Black, E. and El-Awaisi, A. | 2015 | No relevant intervention |
| Thai clinicians' attitudes toward antimicrobial stewardship programs | Sutthiruk, N. and Considine, J. and Hutchinson, A. and Driscoll, A. and Malathum, K. and Botti, M. | 2018 | No relevant intervention |
| The Antimicrobal Stewardship Programme: where have we been...where are we going? | Tsang, J. K. and Tsang, O. T. and Yao, R. and Lai, S. | 2013 | No relevant intervention |
| The current state of antifungal stewardship among pediatric antimicrobial stewardship programs | Eguiguren, L. and Newl and , J. G. and Kronman, M. P. and Hersh, A. L. and Gerber, J. S. and Lee, G. M. and Schwenk, H. T. | 2020 | No relevant intervention |
| The Expanding Role of Antimicrobial Stewardship Programs in Hospitals in the United States: Lessons Learned from a Multisite Qualitative Study | Kapadia, S. N. and Abramson, E. L. and Carter, E. J. and Loo, A. S. and Kaushal, R. and Calfee, D. P. and Simon, M. S. | 2018 | No relevant intervention |
| The impact of pharmacist-led antimicrobial stewardship program on antibiotic use in a county-level tertiary general hospital in China: A retrospective study using difference-in-differences design | Wang, Y. and Zhou, C. and Liu, C. and Liu, S. and Liu, X. and Li, X. | 2022 | No relevant intervention |
| Training family physicians in shared decision making for the use of antibiotics for acute respiratory infections: a pilot clustered randomized controlled trial | Legare, F. and Labrecque, M. and LeBlanc, A. and Njoya, M. and Laurier, C. and Cote, L. and Godin, G. and Thivierge, R. L. and O'Connor, A. and St-Jacques, S. | 2011 | No relevant intervention |
| Using formative evaluation to improve uptake of a web-based tool to support antimicrobial stewardship | Zaidi, S. T. and Thursky, K. A. | 2013 | No relevant intervention |
| What are the challenges for antibiotic stewardship at the community level? An analysis of the drivers of antibiotic provision by informal healthcare providers in rural India | Gautham, M. and Spicer, N. and Chatterjee, S. and Goodman, C. | 2021 | No relevant intervention |
| What influences the implementation and sustainability of antibiotic stewardship programmes in hospitals? A qualitative study of antibiotic pharmacists' perspectives across South West England | Monmaturapoj, T. and Scott, J. and Smith, P. and Watson, M. C. | 2022 | No relevant intervention |
| Who listens and who doesn't? Factors associated with adherence to antibiotic stewardship intervention in a Singaporean tertiary hospital | Wee, L. E. and Chung, S. J. and Tang, S. L. S. and Liew, Y. X. and Tan, L. Y. and Cherng, P. Z. B. and Kwa, L. H. A. and Chlebicki, M. P. | 2020 | No relevant intervention |
| What are the views among pakistani physicians toward antimicrobial resistance and hospital antimicrobial stewardship programs? A multi-site qualitative study | Hayat, K. and Rosenthal, M. and Gillani, A. H. and Zhai, P. and Ji, W. and Chang, J. and Hu, H. and Fang, Y. | 2019 | No relevant intervention |
| Views of Community Pharmacists on Antimicrobial Resistance and Antimicrobial Stewardship in Jordan: A Qualitative Study | Saleh, D. and Abu-Farha, R. and Mukattash, T. L. and Barakat, M. and Alefishat, E. | 2021 | No relevant intervention |
| A Nationwide Survey of Australian General Practitioners on Antimicrobial Stewardship: Awareness, Uptake, Collaboration with Pharmacists and Improvement Strategies | Saha, S. K. and Kong, D. C. M. and Thursky, K. and Mazza, D. | 2020 | No relevant outcome |
| A Randomized Controlled Trial of an Electronic Clinical Decision Support Tool for Inpatient Antimicrobial Stewardship | Ridgway, J. P. and Robicsek, A. and Shah, N. and Smith, B. A. and Singh, K. and Semel, J. and Acree, M. E. and Grant, J. and Ravich and ran, U. and Peterson, L. R. | 2021 | No relevant outcome |
| Acceptability of Proposed Stewardship Interventions to Reduce Preoperative Screening and Treatment of Asymptomatic Bacteriuria | Dukes, K. and Walhof, J. and Brown, M. and Gupta, K. and Strymish, J. and Suh, D. and Alex and er, B. and Au, V. and Beck, B. and Richardson, K. and O'Brien, W. and Chan, J. and Reisinger, H. S. and Schweizer, M. | 2021 | No relevant outcome |
| Acceptance and Outcome of Interventions in Meropenem De-escalation ASP in Pediatrics | Rungsitsathian, K. and Wacharachaisurapol, N. and Nakaranurack, C. and Usayaporn, S. and Sakares, W. and Kawichai, S. and Jantarabenjakul, W. and Puthanakit, T. and Anugulruengkitt, S. | 2021 | No relevant outcome |
| Adherence to recommendations of an antimicrobial stewardship programme | Martin, L. and Murillas, J. and Campins, A. and Penar and a, M. and Perianez, L. and Oliver, A. and Delgado, O. and Riera, M. | 2012 | No relevant outcome |
| Analysis of an Antibiotic Stewardship Program for Asymptomatic Bacteriuria in the Veterans Affairs Health Care System | Grigoryan, L. and Naik, A. D. and Lichtenberger, P. and Graber, C. J. and Patel, P. K. and Drekonja, D. M. and Gauthier, T. P. and Shukla, B. and Sales, A. E. and Krein, S. L. and Van, J. N. and Dillon, L. M. and Hysong, S. J. and Kramer, J. R. and Walder, A. and Ramsey, D. and Trautner, B. W. | 2022 | No relevant outcome |
| Antibiotic assessment at hospital discharge-Room for stewardship intervention | Barnett, S. G. and Lata, P. and Kavalier, M. and Crnich, C. and Balasubramanian, P. | 2020 | No relevant outcome |
| Antibiotic prescriptions in French day-care centres: 1999-2008 | Dunais, B. and van Dijken, C. and Bruno, P. and Touboul, P. and Carsenti-Dellamonica, H. and Pradier, C. | 2011 | No relevant outcome |
| Antibiotic stewardship interventions significantly improve preferred antibiotic prophylaxis in total joint Arthroplasty | Quartuccio, K. and Roberts, R. and Pillinger, K. E. and Heintz, E. and Stern, J. and Myers, T. | 2020 | No relevant outcome |
| Asymptomatic bacteriuria: Impact of an antimicrobial stewardship bundle to reduce unnecessary antibiotics in patients without urinary catheters | Rico, M. and Sulaiman, R. and MacLeod, R. | 2021 | No relevant outcome |
| Clinical impact of a pharmacist-led antimicrobial stewardship initiative evaluating patients with Clostridioides difficile colitis | Bishop, P. A. and Isache, C. and McCarter, Y. S. and Smotherman, C. and Gautam, S. and Jankowski, C. A. | 2020 | No relevant outcome |
| Cost-effectiveness of internet-based training for primary care clinicians on antibiotic prescribing for acute respiratory tract infections in Europe | Oppong, R.; Smith, R. D.; Little, P.; Verheij, T.; Butler, C. C.; Goossens, H.; Coenen, S.; Jowett, S.; Roberts, T. E.; Achana, F.; Stuart, B.; Coast, J.; | 2018 | No relevant outcome |
| Cost-effectiveness of point-of-care C-reactive protein tests for respiratory tract infection in primary care in England | Hunter, R.; | 2015 | No relevant outcome |
| Doctors' Perceptions, Attitudes and Practices towards the Management of Multidrug-Resistant Organism Infections after the Implementation of an Antimicrobial Stewardship Programme during the COVID-19 Pandemic | Spernovasilis, N. and Ierodiakonou, D. and Spanias, C. and Mathioudaki, A. and Ioannou, P. and Petrakis, E. C. and Kofteridis, D. P. | 2021 | No relevant outcome |
| Half of Prescribed Antibiotics Are Not Needed: A Pharmacist-Led Antimicrobial Stewardship Intervention and Clinical Outcomes in a Referral Hospital in Ethiopia | Gebretekle, G. B. and Haile Mariam, D. and Abebe Taye, W. and Mulu Fentie, A. and Amogne Degu, W. and Alemayehu, T. and Beyene, T. and Libman, M. and Gedif Fenta, T. and Yansouni, C. P. and Semret, M. | 2020 | No relevant outcome |
| Practical Pharmacist-Led Interventions to Improve Antimicrobial Stewardship in Ghana, Tanzania, Uganda and Zambia | Kerr, F. and Sefah, I. A. and Essah, D. O. and Cockburn, A. and Afriyie, D. and Mahungu, J. and Mirfenderesky, M. and Ankrah, D. and Aggor, A. and Barrett, S. and Brayson, J. and Muro, E. and Benedict, P. and Santos, R. and Kanturegye, R. and Onegwa, R. and Sekikubo, M. and Rees, F. and B and a, D. and Kalungia, A. C. and Alutuli, L. and Chikatula, E. and Ashiru-Oredope, D. | 2021 | No relevant outcome |
| Survey of physician and pharmacist steward perceptions of their antibiotic stewardship programs | Burrowes, S. A. B. and Drainoni, M. L. and Tjilos, M. and Butler, J. M. and Damschroder, L. J. and Goetz, M. B. and Madaras-Kelly, K. and Reardon, C. M. and Samore, M. H. and Shen, J. and Stenehjem, E. and Zhang, Y. and Barlam, T. F. | 2021 | No relevant outcome |
| The 2018 Lebanese Society of Infectious Diseases and Clinical Microbiology Guidelines for the use of antimicrobial therapy in complicated intra-abdominal infections in the era of antimicrobial resistance | Haddad, N. and Kanj, S. S. and Awad, L. S. and Abdallah, D. I. and Moghnieh, R. A. | 2019 | No relevant outcome |
| The Antibiotic Guardian campaign: a qualitative evaluation of an online pledge-based system focused on making better use of antibiotics | Kesten, J. M. and Bhattacharya, A. and Ashiru-Oredope, D. and Gobin, M. and Audrey, S. | 2017 | No relevant outcome |
| Addressing social influences reduces antibiotic duration in complicated abdominal infection: a mixed methods study | Broom, J. and Tee, C. L. and Broom, A. and Kelly, M. D. and Scott, T. and Grieve, D. A. | 2019 | No relevant population |
| Change the habit to change the practice: Do audits really ever change anything? | Bodansky, D. and Oskrochi, Y. and Judah, G. and Lewis, M. and Fischer, B. and Narayan, B. | 2017 | No relevant population |
| Cost-effectiveness of decision support strategies for safely reducing antibiotic use in acute bronchitis | Michaelidis, C. I.; Kern, M. S.; Smith, K. J | 2014 | No relevant population |
| Antimicrobial prescribing in primary care: An evaluation of factors influencing prescribing and General Practitioners' reported use of strategies to reduce overprescribing | Sin, C. and White, S. and Batchelor, A. and Chapman, S. | 2016 | Not an original study |
| Prescribers' experience and opinions on antimicrobial stewardship programmes in hospitals: a French nationwide survey | Perozziello, A. and Lescure, F. X. and Truel, A. and Routelous, C. and Vaillant, L. and Yazdanpanah, Y. and Lucet, J. C. and group, Cefeca study | 2019 | Not an original study |
| The Global Alliance for Infections in Surgery: defining a model for antimicrobial stewardship-results from an international cross-sectional survey | Sartelli, M. and Labricciosa, F. M. and Barbadoro, P. and Pagani, L. and Ansaloni, L. and Brink, A. J. and Carlet, J. and Khanna, A. and Chichom-Mefire, A. and Coccolini, F. and Di Saverio, S. and May, A. K. and Viale, P. and Watkins, R. R. and Scudeller, L. and Abbo, L. M. and Abu-Zidan, F. M. and Adesunkanmi, A. K. and Al-Dahir, S. and Al-Hasan, M. N. and Alis, H. and Alves, C. and Araujo da Silva, A. R. and Augustin, G. and Bala, M. and Barie, P. S. and Beltran, M. A. *et al.* | 2017 | Not an original study |
| The Perceptions of Health Care Providers Regarding the Implementation of Antimicrobial Stewardship Programs | Ahmed, N. J. and Alrawili, A. S. and Alkhawaja, F. Z. and Haseeb, A. and Hassali, A. A. and Khan, A. H. | 2021 | STUDY NOT AVAILAVLE |
| A survey of physicians' knowledge, attitudes and perceptions about the antimicrobial stewardship programme in a Belgian university hospital with the aim to identify barriers and facilitators of guideline adherence | Capiau, A. and Boelens, J. and Callens, S. and Commeyne, S. and De Waele, J. and Somers, A. and Vogelaers, D. and Buyle, F. | 2020 | Wrong publication type |
| Alert stickers as prescribing aids to limit duration of antimicrobial treatment | Collins, C. and Bradley, K. and Moore, T. and Foley, S. and Fitzpatrick, F. and Smyth, E. G. | 2010 | Wrong publication type |
| An antimicrobial stewardship public commitment poster intervention to improve antibiotic prescribing in a University primary care clinic | Bergman, S. J. and Williams, S. and Alu, C. and Mathews, W. | 2017 | Wrong publication type |
| Antibiotic prescribing patterns in the emergency department for uncomplicated urinary tract infections | DeGeorge, L. and Gardner, S. M. and Page, E. B. and Izzo, J. and Wilson, M. | 2018 | Wrong publication type |
| Antibiotics Smart Use: a workable model for promoting the rational use of medicines in Thailand | Sumpradit, N. and Chongtrakul, P. and Anuwong, K. and Pumtong, S. and Kongsomboon, K. and Butdeemee, P. and Khonglormyati, J. and Chomyong, S. and Tongyoung, P. and Losiriwat, S. and Seesuk, P. and Suwanwaree, P. and Tangcharoensathien, V. | 2012 | Wrong publication type |
| Antimicrobial stewardship intervention bundle decreases fluoroquinolone prescribing for urinary tract infection in urgent care and primary care clinics | Cubillos, A. L. and Caulder, E. and Patch, M. and Saunders, M. B. and Lynch, L. W. and Gieselman, M. R. and Calkins, K. | 2020 | Wrong publication type |
| Antimicrobial stewardship rounds in a general hospital in Ireland | Galvin, M. and Fennell, J. | 2012 | Wrong publication type |
| Antimicrobial stewardship: A review of audit and feedback systems and evaluation of outcomes in a medical center in Taiwan | Chang, Y. W. and Chen, I. L. and Su, L. H. and Lee, C. H. | 2015 | Wrong publication type |
| Asia Pacific antimicrobial stewardship preceptorship program developed and implemented by a medical center in Taiwan | Wang, G. and Hsiao, H. L. and Cheng, C. W. and Chiu, C. H. and Huang, C. T. and Lee, M. H. and Huang, P. Y. and Tsai, T. C. and Chan, Y. Y. and Deng, S. T. and Chang, P. Y. and Wu, T. L. and Wu, T. S. | 2020 | Wrong publication type |
| Comparison of active versus passive strategies in improving compliance to antimicrobial stewardship interventions | Tang, S. S. L. and Zhou, Y. P. and Loo, L. and Kwa, A. L. and Chlebicki, P. | 2018 | Wrong publication type |
| Develop and Implement a Novel Pediatric Antimicrobial Stewardship Program in a Non-Freestanding Children's Hospital Located in an Adult-Centered Community Hospital in San Joaquin Valley, California | Lien, T. C. and Covarrubias, L. and Ip, A. and Husted, H. and Suzuki, E. and Rongkavilit, C. | 2021 | Wrong publication type |
| Effect of academic detailing with prescribers as an antimicrobial stewardship intervention in solid organ transplant patients | So, M. and Morris, A. and Bell, C. and Humar, A. and Husain, S. | 2017 | Wrong publication type |
| Evaluating use of the RPS antibiotic checklist by community pharmacists and its potential impact on the Government's antimicrobial resistance strategy | Hawksworth, G. and Liaqat, A. and Nasar, H. and Qasim, M. and Shiraz, A. and Afzal, S. and Frank, S. and Howard, P. | 2019 | Wrong publication type |
| Implementation Methods for a Collaborative Pharmacist-Led Antimicrobial Stewardship Intervention at Hospital Discharge | Medler, C. and Mercuro, N. and MacDonald, N. and Weinmann, A. and Neuhauser, M. and Hicks, L. and Srinivasan, A. and Divine, G. and Zervos, M. and Davis, S. | 2020 | Wrong publication type |
| Improving antibiotic stewardship on general surgical wards | Clark, M. and Crumley, A. and Wilson, M. | 2021 | Wrong publication type |
| Knowledge, attitude and perception survey of doctors regarding antibiotic use and resistance in Karachi, Pakistan | Khanum, I. and Ahmed, H. and Bhimani, S. | 2019 | Wrong publication type |
| Missed opportunities for shared decision making in antimicrobial stewardship: The potential consequences of a lack of patient engagement in secondary care | Rawson, T. M. and Moore, L. S. P. and Hern and ez, B. and Castro-Sanchez, E. and Charani, E. and Ahmad, R. and Holmes, A. H. | 2016 | Wrong publication type |
| Physician perceptions and attitudes towards an antimicrobial stewardship program and computerized decision-making systems: A focus group study | Chua, A. Q. and Kong, S. T. and Lee, L. W. and Yii, D. Y. C. and Tang, S. S. L. and Lee, W. and Kwa, A. L. and Lie, D. | 2017 | Wrong publication type |
| Primary care clinicians' perceptions about antibiotic prescribing for acute bronchitis: A qualitative study | Linder, J. A. and Dempsey, P. P. and Businger, A. C. and Whaley, L. E. | 2012 | Wrong publication type |
| Re-imagining patient involvement in infection prevention and control and antimicrobial stewardship | Seale, H. and Mitchell, B. and Konecny, P. and Broom, A. and Maley, M. and Torda, A. | 2019 | Wrong publication type |
| Shared decision support for patients. An antimicrobial stewardship strategy to promote appropriate antibiotics use in primary care | Biezen, R. and Manski-Nankervis, J. A. and Somasundaram, K. and Buising, K. | 2021 | Wrong publication type |
| Social media for stewardship: Progress or a waste of time? | Goff, D. | 2014 | Wrong publication type |
| Sustainability of antibiotic stewardship programs: Perceptions & experiences of nursing home staff | Seshadri, S. and Felsen, C. B. and Sellers, C. R. and Dumyati, G. | 2020 | Wrong publication type |
| The barriers and facilitators to optimal antimicrobial prescribing: A qualitative study | Charani, E. and Shah, N. and Edwards, R. and Drumright, L. and Holmes, A. | 2012 | Wrong publication type |
| The impact of a national antimicrobial stewardship programmes on antibiotic prescribing in primary care in England: An interrupted time series analysis | Balinskaite, V. and Holmes, A. and Johnson, A. and Aylin, P. | 2018 | Wrong publication type |
| Using health literacy techniques to develop patient information for counselling on antibiotics courses | Hawksworth, G. and Beck, K. and Ioannou, K. and Ahmed, S. and Zaman, S. and Afzal, S. and Frank, S. and Howard, P. | 2020 | Wrong publication type |
| Using health literacy techniques to support pharmacist practice when counselling on antibiotics courses | Frank, S. and Hawksworth, G. and Beck, K. and Ioannou, K. and Ahmed, S. and Zaman, S. and Afzal, S. and Howard, P. | 2020 | Wrong publication type |
| What determines the patient's receipt of antibiotics recommended by computerised decision support systems? | Chow, A. and Lye, D. | 2015 | Wrong publication type |
| Why don't doctors accept recommendations by antibiotic computerised decision support systems?: A mixed methods study | Chow, A. and Lye, D. C. B. | 2014 | Wrong publication type |
| An antibiotic stewardship programme to reduce inappropriate antibiotic prescribing for acute respiratory infections in rural Chinese primary care facilities: study protocol for a clustered randomised controlled trial | Zhuo, C. and Wei, X. and Zhang, Z. and Hicks, J. P. and Zheng, J. and Chen, Z. and Haldane, V. and Walley, J. and Guan, Y. and Xu, H. and Zhong, N. | 2020 | Wrong study design |
| ATOUM 6: does a multimodal intervention involving nurses reduce the use of antibiotics in French nursing homes? | Ahouah, M. and Lombrail, P. and Gavazzi, G. and Chaaban, T. and Rothan-Tondeur, M. | 2019 | Wrong study design |
| Development of a patient-centred intervention to improve knowledge and understanding of antibiotic therapy in secondary care | Rawson, T. M. and Moore, L. S. P. and Castro-Sanchez, E. and Charani, E. and Hern and ez, B. and Alividza, V. and Husson, F. and Toumazou, C. and Ahmad, R. and Georgiou, P. and Holmes, A. H. | 2018 | Wrong study design |
| Improving shared decision-making in adolescents through antibiotic education | Ngadimon, I. W. and Islahudin, F. and Mohamed Shah, N. and Md Hatah, E. and Makmor-Bakry, M. | 2017 | Wrong study design |
| The protocol of improving safe antibiotic prescribing in telehealth: A randomized trial | McCabe, B. K. and Linder, J. A. and Doctor, J. N. and Friedberg, M. and Fox, C. R. and Goldstein, N. J. and Knight, T. K. and Kaiser, K. and Tibbels, J. and Haenchen, S. and Persell, S. D. and Warberg, R. and Meeker, D. | 2022 | Wrong study design |
| Written information for patients (or parents of child patients) to reduce the use of antibiotics for acute upper respiratory tract infections in primary care | O'Sullivan, J. W. and Harvey, R. T. and Glasziou, P. P. and McCullough, A. | 2016 | Wrong study design |

# Appendix 3: Quality Assessment (Using Mixed Methods Appraisal Tool)

|  | Qualitative | | | | | Quantitative randomized controlled trials | | | | | Quantitative nonrandomized (that is, Non-RCT comparative studies) | | | | | Quantitative descriptive | | | | | Mixed methods | | | | |
| --- | --- | --- | --- | --- | --- | --- | --- | --- | --- | --- | --- | --- | --- | --- | --- | --- | --- | --- | --- | --- | --- | --- | --- | --- | --- |
| Study ID (Author, Year) | 1.1 | 1.2 | 1.3 | 1.4 | 1.5 | 2.1 | 2.2 | 2.3 | 2.4 | 2.5 | 3.1 | 3.2 | 3.3 | 3.4 | 3.5 | 4.1 | 4.2 | 4.3 | 4.4 | 4.5 | 5.1 | 5.2 | 5.3 | 5.4 | 5.5 |
| Ackerman, 2013^1^ | Y | Y | Y | Y | Y |  |  |  |  |  |  |  |  |  |  | Y | N | Y | Y | Y | Y | Y | Y | Y | Y |
| Agency For Healthcare Research and Quality, 2022^2^ |  |  |  |  |  |  |  |  |  |  | Y | Y | Y | CT | Y |  |  |  |  |  |  |  |  |  |  |
| Allison, 2020^3^ |  |  |  |  |  |  |  |  |  |  |  |  |  |  |  | Y | Y | Y | Y | CT |  |  |  |  |  |
| Andreeva, 2014^4^ |  |  |  |  |  | Y | Y | Y | N | CT |  |  |  |  |  |  |  |  |  |  |  |  |  |  |  |
| Anthierens, 2015^5^ | Y | Y | Y | Y | Y |  |  |  |  |  |  |  |  |  |  |  |  |  |  |  |  |  |  |  |  |
| Ashiru-Oredope, 2020^6^ |  |  |  |  |  | CT | CT | CT | Y | CT |  |  |  |  |  |  |  |  |  |  |  |  |  |  |  |
| Avent, 2024^7^ |  |  |  |  |  | Y | Y | Y | CT | Y |  |  |  |  |  |  |  |  |  |  |  |  |  |  |  |
| Biezen, 2021(NCAS)^8^ Australia | Y | Y | Y | Y | Y |  |  |  |  |  |  |  |  |  |  |  |  |  |  |  |  |  |  |  |  |
| Bjerrum, 2011 ^9^ |  |  |  |  |  |  |  |  |  |  | CT | Y | Y | CT | Y |  |  |  |  |  |  |  |  |  |  |
| Butler, 2012^10^ |  |  |  |  |  | Y | Y | Y | CT | Y |  |  |  |  |  |  |  |  |  |  |  |  |  |  |  |
| Cals, 2010^11^ |  |  |  |  |  | Y | Y | Y | N | Y |  |  |  |  |  |  |  |  |  |  |  |  |  |  |  |
| Cals, 2013^12^ |  |  |  |  |  | CT | Y | Y | CT | Y |  |  |  |  |  |  |  |  |  |  |  |  |  |  |  |
| Chiswell, 2019^13^ |  |  |  |  |  |  |  |  |  |  | Y | Y | CT | Y | CT |  |  |  |  |  |  |  |  |  |  |
| Chung, 2017^14^ | Y | Y | Y | Y | Y |  |  |  |  |  |  |  |  |  |  |  |  |  |  |  |  |  |  |  |  |
| Cross, 2019^15^ |  |  |  |  |  | CT | CT | Y | CT | CT |  |  |  |  |  |  |  |  |  |  |  |  |  |  |  |
| Dekker, 2018^16^ |  |  |  |  |  | Y | Y | Y | CT | Y |  |  |  |  |  |  |  |  |  |  |  |  |  |  |  |
| Dekker, 2019^17^ |  |  |  |  |  | CT | Y | CT | CT | CT |  |  |  |  |  |  |  |  |  |  |  |  |  |  |  |
| Eley, 2018^18^ | Y | Y | Y | Y | Y |  |  |  |  |  |  |  |  |  |  |  |  |  |  |  |  |  |  |  |  |
| Eley, 2020^19^ | Y | Y | Y | Y | Y |  |  |  |  |  |  |  |  |  |  | Y | CT | Y | CT | Y | CT | Y | Y | CT | CT |
| Forrest, 2022^20^ |  |  |  |  |  |  |  |  |  |  | CT | Y | CT | CT | CT |  |  |  |  |  |  |  |  |  |  |
| Francis, 2013^21^ | Y | Y | Y | Y | Y |  |  |  |  |  |  |  |  |  |  |  |  |  |  |  |  |  |  |  |  |
| Francis, 2020^22^ |  |  |  |  |  | Y | Y | Y | Y | Y |  |  |  |  |  |  |  |  |  |  |  |  |  |  |  |
| Giry, 2016^23^ |  |  |  |  |  |  |  |  |  |  |  |  |  |  |  | Y | CT | Y | N | Y |  |  |  |  |  |
| Goggin, 2022^24^ |  |  |  |  |  | CT | Y | Y | CT | Y |  |  |  |  |  |  |  |  |  |  |  |  |  |  |  |
| Gonzales, 2013^25^ |  |  |  |  |  | CT | Y | Y | CT | Y |  |  |  |  |  |  |  |  |  |  |  |  |  |  |  |
| Gulliford, 2014^26^ |  |  |  |  |  | Y | CT | Y | CT | Y |  |  |  |  |  |  |  |  |  |  |  |  |  |  |  |
| Hernandez-Santiago, 2015^27^ |  |  |  |  |  |  |  |  |  |  | Y | Y | Y | Y | Y |  |  |  |  |  |  |  |  |  |  |
| Hounkpatin, 2021^28^ | Y | Y | Y | Y | Y |  |  |  |  |  |  |  |  |  |  |  |  |  |  |  |  |  |  |  |  |
| Huddy, 2016^29^ | Y | Y | Y | Y | Y |  |  |  |  |  |  |  |  |  |  |  |  |  |  |  |  |  |  |  |  |
| Jenkins, 2013^30^ |  |  |  |  |  | Y | CT | Y | CT | Y |  |  |  |  |  |  |  |  |  |  |  |  |  |  |  |
| Jones, 2017^31^ | Y | Y | Y | Y | Y |  |  |  |  |  |  |  |  |  |  | CT | Y | CT | Y | CT | CT | CT | CT | CT | CT |
| Legare, 2012^32^ |  |  |  |  |  | Y | Y | CT | Y | CT |  |  |  |  |  |  |  |  |  |  |  |  |  |  |  |
| Lemiengre, et al 2018^33^ |  |  |  |  |  | Y | Y | Y | CT | Y |  |  |  |  |  |  |  |  |  |  |  |  |  |  |  |
| Lemiengre, 2018^34^ |  |  |  |  |  | CT | N | Y | CT | Y |  |  |  |  |  |  |  |  |  |  |  |  |  |  |  |
| Likopa, 2022^35^ |  |  |  |  |  | Y | Y | Y | Y | Y |  |  |  |  |  |  |  |  |  |  |  |  |  |  |  |
| Little, 2019^36^ |  |  |  |  |  | Y | Y | Y | CT | Y |  |  |  |  |  |  |  |  |  |  |  |  |  |  |  |
| Little, 2013^37^ |  |  |  |  |  | Y | Y | Y | N | Y |  |  |  |  |  |  |  |  |  |  |  |  |  |  |  |
| Llor, 2014^38^ |  |  |  |  |  |  |  |  |  |  | CT | CT | Y | CT | Y |  |  |  |  |  |  |  |  |  |  |
| Llor, 2015^39^ |  |  |  |  |  |  |  |  |  |  | CT | CT | Y | CT | Y |  |  |  |  |  |  |  |  |  |  |
| Madaras-Kelly, 2020^40^ |  |  |  |  |  |  |  |  |  |  | Y | Y | Y | CT | Y |  |  |  |  |  |  |  |  |  |  |
| McDermott, 2014^41^ | Y | Y | Y | Y | Y |  |  |  |  |  |  |  |  |  |  | CT | Y | Y | Y | Y | Y | Y | Y | Y | Y |
| McIsaac, 2021^42^ |  |  |  |  |  |  |  |  |  |  | CT | CT | Y | CT | CT |  |  |  |  |  |  |  |  |  |  |
| Meeker, 2014^43^ |  |  |  |  |  | Y | Y | Y | CT | Y |  |  |  |  |  |  |  |  |  |  |  |  |  |  |  |
| Milos, 2013^44^ |  |  |  |  |  | CT | N | N | CT | CT |  |  |  |  |  |  |  |  |  |  |  |  |  |  |  |
| Mowbray, 2020^45^ | Y | Y | Y | Y | Y |  |  |  |  |  |  |  |  |  |  |  |  |  |  |  |  |  |  |  |  |
| Muhia, 2016^46^ |  |  |  |  |  |  |  |  |  |  |  |  |  |  |  | Y | Y | Y | Y | Y |  |  |  |  |  |
| Patel, 2022^47^ |  |  |  |  |  |  |  |  |  |  |  |  |  |  |  | CT | CT | Y | N | Y |  |  |  |  |  |
| Peters, 2013^48^ |  |  |  |  |  |  |  |  |  |  | Y | Y | Y | Y | Y |  |  |  |  |  |  |  |  |  |  |
| Pittenger, 2015^49^ |  |  |  |  |  |  |  |  |  |  | Y | Y | Y | Y | Y |  |  |  |  |  |  |  |  |  |  |
| PoB-Doering, Dec 2020^50^ | Y | Y | Y | Y | Y |  |  |  |  |  |  |  |  |  |  | Y | Y | Y | Y | Y | Y | Y | Y | CT | Y |
| Poss-Doering, 2020^51^ | Y | Y | Y | Y | Y |  |  |  |  |  |  |  |  |  |  |  |  |  |  |  |  |  |  |  |  |
| Poss-Doering, Oct 2020^52^ | Y | Y | Y | Y | Y |  |  |  |  |  |  |  |  |  |  | Y | Y | Y | Y | Y | Y | Y | Y | CT | Y |
| Sloane, 2020^53^ |  |  |  |  |  |  |  |  |  |  | Y | CT | Y | CT | Y |  |  |  |  |  |  |  |  |  |  |
| Tonkin-Crine, 2023^54^ | Y | Y | Y | Y | Y |  |  |  |  |  |  |  |  |  |  | Y | CT | Y | Y | Y | CT | Y | CT | Y | Y |
| Tonna, 2020^55^ | Y | Y | Y | CT | Y |  |  |  |  |  |  |  |  |  |  |  |  |  |  |  |  |  |  |  |  |
| van Esch, 2018^56^ |  |  |  |  |  |  |  |  |  |  |  |  |  |  |  | Y | Y | Y | N | Y |  |  |  |  |  |
| Wei, 2017^57^ |  |  |  |  |  | Y | Y | CT | Y | CT |  |  |  |  |  |  |  |  |  |  |  |  |  |  |  |
| Wei, 2019^58^ |  |  |  |  |  | Y | Y | Y | N | Y |  |  |  |  |  |  |  |  |  |  |  |  |  |  |  |
| Yardley, 2013^59^ |  |  |  |  |  | Y | CT | CT | N | CT |  |  |  |  |  |  |  |  |  |  |  |  |  |  |  |

Y= yes; N = No; CT = Can’t tell.

| 1.1 Is the qualitative approach appropriate to answer the research question? |
| --- |
| 1.2 Are the qualitative data collection methods adequate to address the research question? |
| 1.3 Are the findings adequately derived from the data? |
| 1.4 Is the interpretation of results sufficiently substantiated by data? |
| 1.5 Is there coherence between qualitative data sources, collection, analysis and interpretation? |
| 2.1 Is randomization appropriately performed? |
| 2.2 Are the groups comparable at baseline? |
| 2.3 Are there complete outcome data? |
| 2.4 Are outcome assessors blinded to the intervention provided? |
| 2.5 Did the participants adhere to the assigned intervention? |
| 3.1 Are the participants representative of the target population? |
| 3.2 Are measurements appropriate regarding both the outcome and intervention (or exposure)? |
| 3.3 Are there complete outcome data? |
| 3.4 Are the confounders accounted for in the design and analysis? |
| 3.5 During the study period, is the intervention administered (or exposure occurred) as intended? |
| 4.1 Is the sampling strategy relevant to address the research question? |
| 4.2 Is the sample representative of the target population? |
| 4.3 Are the measurements appropriate? |
| 4.4 Is the risk of nonresponse bias low? (Say yes if response rate higher than 60%) |
| 4.5 Is the statistical analysis appropriate to answer the research question? |
| 5.1 Is there an adequate rationale for using a mixed methods design to address the research question? |
| 5.2 Are the different components of the study effectively integrated to answer the research question? |
| 5.3 Are the outputs of the integration of qualitative and quantitative components adequately interpreted? |
| 5.4 Are divergences and inconsistencies between quantitative and qualitative results adequately addressed? |
| 5.5 Do the different components of the study adhere to the quality criteria of each tradition of the methods involved? |

# Appendix 4: Interventions

| **Intervention name  Country Study ID** | **Rationale** | **What was provided (healthcare professional's and patients components)** | **Who provided the intervention and how it was provided** | **Mode of delivery and where the intervention occurred** | **When and How Much** | **Tailoring and modifications** | **How well** |
| --- | --- | --- | --- | --- | --- | --- | --- |
| Antibiotic Review Kit (ARK) UK Cross ,2019^15^ | To support prescribing and non-prescribing healthcare professionals to apply the ‘review and revise’ approach to antibiotic prescriptions for acute and general medical inpatients, and specifically to stop antibiotic treatment more often when it is safe to do so | ARK has four components: an online tool; prescribing decision aid; data collection and feedback process; and a patient leaflet | ARK Champion and Core Team | Online | NR | NR | Feedback process |
| Antibiotic Stewardship Training and Quality Improvement Intervention (Bespoke) USA Sloane 2020^53^ | Reducing inappropriate overprescribing and, ultimately, of slowing the development of resistant bacteria and the occurrence of infections with MDROs | Intervention components included: an optional standardized system for recording and reporting antibiotic prescribing; two 1-hour in-service training modules for nurses; two 1-hour case-discussion audiocasts on CD-ROM mailed to all medical providers; posters for placement within NHs; pocket information and reminder cards containing an algorithm to guide staff; pocket cards for medical providers; periodic quality improvement reports to nurses and medical directors; an informational brochure for residents and families; and free internet access to training modules and continuing education credit. | US Centres for Medicare and Medicaid Services (CMS) | Online and offline-such as training materials, Audiocast, posters, reminder card, brochures | Two of each 1-hour in-service training and 1 hour audio case discussion | NR | NR |
| ARena study Germany Poss-Doering 2020^51^ | To address physician, ambulatory care team and patient knowledge and attitudes about the use of antibiotics | Each arm received a different set of intervention components comprised of e-learning on communication, quality circles and data-based feedback for physicians and nonphysician health professionals, information campaigns for the public, performance-based additional reimbursement, a computerized decision support system and culture sensitive information material for patients in print and digital format on tablet computers to be used in waiting areas. | Study team | e-learning on communication and data-based feedback for physician, computerized decision support system and digital information material in waiting areas for patients | NR | NR | NR |
| ARK-Hospital intervention-GRACE-INTRO UK Mowbray, 2020^45^ | To safely reduce antibiotic use through increasing effective implementation of ‘review and revise’, focusing on facilitating stopping antibiotics when they are no longer needed. | Patient education leaflet provides patients with brief information about when antibiotics are used, the possible risks of taking antibiotics, the ‘review and revise’ process and advice about what to do when their antibiotics are stopped. | Study team | Digital | A 30 min personalized digital education module for self-completion. Regular 45–60 min peer-led seminars for discussing experiences of ‘review and revise’ | Stakeholder consultation | A short quiz added to the online tool for feedback. |
| Bespoke AMS France Giry, 2016^23^ | To assess the acceptability of antibiotic stewardship measures | Questionnaire which comprises of socio-demographic data; evaluation of the acceptability of the 31 measures selected | French Ministry of Health | Online-Anywhere | Flexible- at own pace | NR | NR |
| Bespoke AMS multi-country Little, 2019^36^ | To explore patients’ concerns and to reduce antibiotic prescribing | In the CRP arm, practices were given Internet-based training on use of a CRP point-of-care test. In the communication arm, practices were given Internet-based training on enhanced communication skills and use of an interactive patient booklet. | Study team | Internet based and face to face CRP | NR | NR | NR |
| Bespoke AMS USA Patel, 2022^47^ | (1) to not interfere with clinician workflows at the point of care (e.g., introducing a hard stop in the electronic health record if only a nonantibiotic appropriate diagnosis was documented during a patient visit), (2) to not cause undesirable behaviour change like diagnosis shifting, and (3) to not decrease patient satisfaction. | A. Clinician comparative feedback and education meant for the clinician. Education part includes- 1. to view an internally developed 3-minute video showcasing a physician patient interaction. 2. Presentation by subject-matter expert-A physician champion also conducted a series of educational sessions discussing appropriate management of viral illnesses for urgent care clinicians.  B. Patient education consist of: - 1. Commitment posters. 2. Patient education pamphlets | Study team | NR | Includes a 3-minute video showcasing a physician– patient interaction | NR | NR |
| Bespoke AMS USA Forrest, 2020^20^ | To promote evidence-based practice preventing misuse of resources. T | Four core interventions consists of biweekly QI team meetings, Patient engagement SDA, Antibiotic prescribing 5 Ds tool (Antibiotic prescribing Stewardship Checklist), and a retrospective Case Management Log | Study team | NR | Over 90 days | A provider resource based on the “5 Ds of Antimicrobial Stewardship Checklist” was adapted from the Public Health Ontario (2017) clinical reference tool, | QI meetings |
| Bespoke AMS Sweden Milos, 2013^44^ | To reinforce GP’s confidence in their ability to manage URTIs without antibiotics.  To influence the GP’s belief about the positive consequences of managing URTIs without prescribing an antibiotic (OLT and SCT) | Questionnaire-based behaviour change interventions for both intervention and control group. First intervention group additionally received the | Swedish Medical Products Agency, | Posted letter | NR | Questionnaire-based behaviour change interventions that had been validated in a previous experimental study were translated into Swedish, back-translated into English for verification and sent to the GPs by mail. | NR |
| Bespoke AMS Scotland, UK Tonna, 2020^55^ | “To increase awareness of global antibiotic resistance and to encourage best practices among the general public, health workers and policy makers to avoid the further emergence and spread of antibiotic resistance | Information leaflets for patients which is to be given by the pharmacists | A research team of pharmacists with expertise in AMS, community pharmacy practice and qualitative research was established to oversee the study. | Face to face at pharmacy | NR | NR | NR |
| Bespoke AMS China Wei, 2017^57^ | To change doctors’ antibiotic prescribing behaviour for childhood upper respiratory tract infections | A 2-hr interactive training session integrated within routine training procedures. Also includes leaflets and a video educating caregivers about antibiotics. The leaflets were provided to parents and caregivers by doctors during consultations, and the educational video was played on a loop in the hospital waiting areas. | Guangxi Centre for Disease Control and Prevention (CDC; Nanning, China), and colleagues in Rong County and Liujiang County CDCs who coordinated the trial implementation | NR | A 2-h interactive training session for a month | NR | By monthly peer review meetings, and monthly administrative meetings, |
| Bespoke AMS USA Chiswell, 2019^13^ | Reducing immediate antibiotic prescribing in RTIs in a rural setting PC clinic. | Patient education materials, posters and videos. | Researcher following the clinical practice guidelines (CPGs) from NICE | Face to face | NR | NR | NR |
| Bespoke AMS USA Jenkins, 2013^30^ | To assist providers in determining whether an antibiotic should be prescribed, the optimal antibiotic choice when indicated, and the shortest appropriate duration of therapy. | One-page decision support algorithm designed to assist providers in determining an antibiotic prescription, the optimal antibiotic choice when indicated, and the shortest appropriate duration of therapy. In addition to the clinical pathways, the intervention consisted of patient education materials. | Study team | NR | NR | NR | NR |
| Bespoke AMS China Wei, 2019^58^ | To change doctors’ antibiotic prescribing behaviour for childhood upper respiratory tract infections. | Two-hr interactive training session was provided for doctors. The training covered use of the guidelines, as well as communication skills and case study-based roleplays. Finally, the intervention includes educational leaflet for caregivers and a video, played on a loop in the hospital waiting area. | Study team | Face to face | Two-h interactive training session within the first month following the implementation of the intervention. | NR | The intervention was designed to be self-sustaining, e.g., prescription peer reviews were first conducted with the trial team and then by each facility during their routine monthly clinician meetings. |
| Bespoke AMS USA Meeker, 2014^43^ | To emphasize clinician commitment to guidelines for appropriate antibiotic prescribing and explained why antibiotics were not appropriate in many cases. | (1) The intervention of a posted commitment letter-The commitment poster condition required clinician photographs and signatures as endorsement on a poster-sized commitment letter (18 × 24 inches) displayed in their examination rooms for a 12-week period beginning in mid-February 2012 or (2) The standard practice control. | Study team | Face-to-face to view a poster-sized commitment letter | A 12-week period in clinical examination room | NR | NR |
| Bespoke AMS USA Ackerman, 2013^1^ | Information about appropriate antibiotic use for cough and cold illness from the U.S. Centres for Disease Control | A print-based and electronic intervention strategies-At print-based practices, the intervention consisted of a poster depicting a diagnostic algorithm for cough illness that was placed in all examination rooms. At EMR-based practices, a similar algorithm was programmed into the health system’s electronic medical record and clinicians were provided with an “order set”. All practices received patient education brochures. Clinician “champions” were also assigned to each practice and invited to attend a training session. | Clinician “champions” | Face to face lecture and small group discussion, education sessions | The sessions were held at the beginning of the intervention period and varied in size from one-on-one to small groups. | NR | Feedback from practitioner |
| Bespoke AMS USA Gonzales, 2013^25^ | To address predisposing factors (clinician education and practice guidelines), reinforcing factors (clinical champions and audit and feedback), and enabling factors (patient education and decision support) | The printed decision support (PDS-using an algorithmic approach) and computer-assisted decision support (CDS) arms both received intervention components guided by the PRECEDE-PROCEED (predisposing, reinforcing, and enabling constructs in educational diagnosis and evaluation–policy, regulatory, and organizational constructs in educational and environmental development) model. Clinician education was delivered for each clinic by a clinical champion. At PDS intervention sites, patient educational brochures were provided by triage nurses to patients, and a poster displaying the clinical algorithm decision support was placed in all examination rooms | Clinical champions | At each clinic | A half-day training session | NR | NR |
| Bespoke AMS multi-country Little, 2013^37^ | To gather of information on patients’ concerns and expectations, exchange of information on symptoms, natural disease course, and treatments, agreement of a management plan, summing up, and providing guidance about when to reconsult. | The CRP group received internet training on how to target testing and how to negotiate with the patient about management decisions. Training in enhanced communication skills focused on the gathering of information on patients’ concerns and expectations, exchange of information on symptoms, natural disease course, and treatments, agreement of a management plan, summing up, and providing guidance about when to reconsult. Physicians were also provided with an interactive booklet to use during consultations. The training was supported by video demonstrations of consultation techniques. The internet modules and materials were translated into the relevant national language and mainly addressed lower respiratory-tract infections. | Study team and manufacturer | Internet training and a repeat audit and onsite training | February to May 2011, the end of the season for respiratory-tract infections | NR | During a run-in period of several weeks before data collection began, physicians practised using the device. |
| Bespoke AMS The Netherlands Cals, 2013^12^ | To inform GP on the additional diagnostic value of CRP in ruling out pneumonia | (1) Physician use of CRP testing, (2) Physician communication skills training, (3) both interventions combined, and (4) usual care. | Study team and an experienced moderator from the Department of Vocational Training in General Practice | Face to face at a central location | Three seminars will be held, with 5–8 GPs participating per session. one two-hour training seminar at a central location for communication training | Practices will get a 8-week run-in period before inclusion starts for familiarisation with the device and interpretation of the results | 8-week run-in period before actual intervention |
| Bespoke AMS Russian Federation Andreeva, 2014^4^ | To combat Resistance against Antibiotics in Community-acquired lower respiratory tract infections in Europe | Before the initiation of the trial, all GPs were provided two vocational training sessions concerning the CRP test (theoretical and practical information). They were given guidelines about the interpretation of CRP results in determining antibiotic prescription. However, the management, including antibiotic treatment, should be decided for each patient on an individual basis. | NR | Vocational training | Two vocational training before the starting of the trial | NR | Two months before the trial, a baseline study without CRP testing was conducted that included 13 of the 18 participating GPs, using the same case report form (CRF) and examination. This allowed observation of prescription rates before and after the clinical trial, serving as a sensitivity analysis |
| Bespoke AMS The Netherlands van Esch, 2018^56^ | (i) Episodes for which antibiotics could be considered (preference-sensitive situations); (ii) episodes for which antibiotics were indicated (should generally be prescribed); and (iii) episodes for which antibiotics were not indicated (should generally not be prescribed). | SDM at the practice level | NR | NR | NR | NR | NR |
| Bespoke AMS UK McDermott, 2014^41^ | To increase GP adherence to guidelines for antibiotic prescribing in primary care. | Section 1: Software: The questions required the GP to rate their level of agreement with statements relating to the way in which prompts could be accessed, read and used during a consultation. Section 2: Prompt type: The questions required the GP to rate their level of agreement with the statement that a prompt was useful in supporting practice (this was conducted for each type of prompt). Section 3: Consultation: This section aimed to assess the ease with which the prompts could be used during a consultation. Section 4: Additional issues: This section combined two topics: agreement with guidelines and communication. Agreement with guidelines: These questions aimed to assess the degree to which GPs were familiar with and agreed with NICE guidelines for the non-prescription of antibiotics in patients with RTI. Communication: These questions aimed to assess the quality of communication relating to the prompts and implementation with GP practices | The intervention content was informed by the UK National Institute for Health and Care Excellence (NICE) | The intervention was installed remotely through a system known as DXS Point-of-Care. | The intervention was activated during consultations with patients who were presenting with a RTI. | The intervention was informed by behaviour change theory and was developed using feedback from qualitative interviews with GPs | The intervention was informed by behaviour change theory and was developed using feedback from qualitative interviews with GPs |
| Bespoke AMS USA Pittenger, 2015^49^ | To provide evidence-based care for patients with ARI, but without unnecessary provider visits and unnecessary antibiotics, | Education on appropriate indications for antibiotic treatment in ARI, tracking, and local benchmarking of provider antibiotic prescription rates. For lower-risk patients, nursing phone care consisted of conﬁrmation of uncomplicated ARI, patient education about the self-limited nature of the disease, and counselling regarding self care . | Study team | Education coaching and feedback | Over 4 months | NR | The quality improvement intervention was developed and implemented using lean principles and encompassed academic detailing and nurse phone care, targeted at root causes of inappropriate antibiotics. |
| Bespoke AMS Australia Biezen, 2021^8^ | To provide education around their diagnosis and how to self-manage their conditions. | Seven patient information sheets. | AMS study team | NR | NR | The co-design sessions provided opportunities for healthcare providers and consumers to discuss the strengths and limitations of existing patient information resources. Together they advised on the attributes of patient information sheets that would address their information needs. | The seven patient information sheets were piloted across eight general practices in metropolitan and regional Victoria between August and November 2020. |
| Bespoke AMS UK Hernandez-Santiago, 2015^27^ | NR | At Health Board level: - Practices received a range of educational material.  At the practice level, practices were asked to: Review the prescribing of antibiotics in the practice. GPs were encouraged to use this to demonstrate quality improvement activity in their annual appraisal. Discuss and agree a policy for telephone prescribing, including how to explain the correct use of antibiotics to patients and how to manage inappropriate requests for antibiotics. | Study team related to Tayside Health Board | NR | NR | NR | NR |
| Bespoke AMS USA Madaras-Kelly, 2020^40^ | NR | Rapid antigen detection test, audit and feedback, For the education and expertise core element, clinicians were encouraged to access vignettes on effective communication strategies for managing ARIs through a multimedia platform, and sites were provided patient educational materials for distribution during visit | Site champions | NR | NR | NR | NR |
| Bespoke AMS USA Muhia, 2016^46^ | To decrease inappropriate antibiotic use in the treatment of respiratory infections by educating primary care providers at an outpatient setting. | There were four education sessions over the project period. In each education session, the DNP student presented current statistics of antibiotics use in RTI, the pros and cons of antibiotic overuse and relevant public health concerns, and the evidence-based delayed antibiotic-prescribing intervention. This education was delivered as a PowerPoint presentation and printed handouts. After the presentation, a skill-building exercise using a case study and guided the discussion were presented. Nurses (RNs) and nurse educators were instructed how to access patient education materials about management of RTIs and how delayed antibiotic prescribing works in the hospital website. Supportive resources such as the NICE guidelines and patient education brochures were posted on the hospital’s website. Providers were given the knowledge test questionnaire twice (before and after the education sessions) to assess their knowledge about the effect of antibiotic overuse and delayed antibiotic prescribing. | DNP students | This education was delivered as a PowerPoint presentation and printed handouts. The education sessions took place in a conference room within the ambulatory care centre | Four one-hour weekly sessions over the project period | NR | NR |
| Bespoke AMS-POC-CRPT UK Tonkin-Crine, 2023^54^ | To support the use of three AMS strategies; enhanced communication skills +/- patient leaflet, delayed prescriptions and POC-CRPT. | Enhanced communication skills +/- patient leaflet, delayed prescriptions and POC-CRPT. The implementation intervention included: 1. Identifying a Champion. 2. Holding a practice meeting to agree a practice-wide approach to implementation. 3. An ‘Antibiotic Optimisation’ website including: o Implementation support for the Champion, o Sections on 3 AMS strategies for clinicians, 4. Physical resources: patient leaflets, POC-CRPT equipment, clinician handouts. | Study team | Remotely via website | NR | NR | NR |
| Bespoke AMS-RAAK The Netherlands Dekker, 2018^16^ | To promote positive expectations and self-confidence in GPs and patients to manage the infection without antibiotics); | The intervention consisted of online training for GPs and a written information booklet for parents. The online training consisted of three parts- 1. a general background about the relevance of prudent antibiotic use and information about antibiotic-related problems, 2. the child-specific parts of the four national RTI guidelines of the Dutch College of GPs, and 3. training in enhanced communication skills, supported by videos of consultation techniques. | Study team | NR | NR | These were adapted from an intervention for adults | Feedback from GPs and patients to ensure that it addressed their concerns and was persuasive) |
| Bespoke PC-AMS Canada McIsaac, 2021^42^ | To reduce antibiotic prescriptions in adults presenting with sore throats (tonsillitis, pharyngitis), acute bronchitis, acute sinusitis, non-specific upper respiratory tract infections (URIs), and acute uncomplicated cystitis. | The multi-faceted intervention included a clinician education program, patient education materials, prescribing decision aids, antibiotic-specific communication skills scripts for patient engagement, and patient advice regarding when to re-consult in cases in which antibiotics were not prescribed (‘safety-netting’) | Clinic champions | NR | The modules took approximately 2 hours to complete and could be completed at the clinician’s convenience | NR | Audit of and feedback on antibiotic prescribing at each intervention clinic was conducted by study team members |
| BISNA and POC-CRP Belgium Lemiengre, 2018^33^ | To reduce antibiotic prescribing for acute respiratory tract infections in adults, | The brief intervention consisted of the following three questions for parents at the start of the consultation: ‘Are you concerned [about the illness of your child]?’, ‘What exactly concerns you?’, and ‘Why does this concern you?’ Apart from these questions, a parent information leaflet containing information about supportive treatment and when to re-consult was provided as safety net advice. | Study team | Face to face | NR | NR | NR |
| CHANGE-3 Germany Poss-Doering, 2020^50^ | To strengthen health literacy competencies in GPs, MAs and patients. | Educational interventions as well as to the public awareness campaign. All practices in the intervention group had access to an e-learning module focusing on strategies for provider patient communication. They also received a tablet pc which was intended to deliver thematically focused information about the use of antibiotics to patients in waiting areas via a pre-installed e-learning application. In addition, a regional intervention ran from September 2018 to January 2020. It consisted of a web- and paper based public awareness campaign addressing children, adolescents, parents, young and middle-aged adults as well as the older population with a multi-media approach using digital and paper-based information material. Printed background information detailing consequences of inappropriate antibiotics use and possible alternatives for treatment of ARTI were provided to the intervention and to the control group. | Experienced outreach visitors (OVs) | e learning and digital, Multimedia and paper based for regional awareness | NR | NR | NR |
| CHANGE-3 Germany Poss-Doering, 2020^52^ | NR | The intervention components focused in this study comprised tablet devices with educational contents for patients, an e-learning platform for medical professionals which offered a communication skills training, and the study-specific website on rational usage of antibiotics for both groups | NR | Multimedia approaches and digital information device | NR | NR |  |
| CRP assistance The Netherlands Cals, 2010^11^ | To help the physician to better triage patients into no prescription, delayed prescription, or immediate prescription strategies without compromising patient outcomes | CRP assistance and no CRP assistance | Study team | Face to face | NR | NR | NR |
| CRP POCT UK Eley, 2018^18^ | NR | Point of care CRP testing, and training | NR | NR | NR | NR | NR |
| CRP POCT Latvia Likopa, 2022^35^ | To find the association with a lower antibiotic prescription rate for adults with respiratory tract infections in primary care | CRP POCT and educational training consists of child with fever – evaluation, precautionary level system and management, child with upper and lower respiratory infection evaluation and management, principles of antibiotic resistance and safer prescribing of antibiotics. This intervention involved one four-hour training seminar, followed by educational materials in video and printed format. FP also received parent information booklets about managing children with fever at home and signs to look out for that indicate a FP should be contacted. | Study team provided educational training and the manufacturer on how to perform the test | Face-to-face meeting, | NR | NR | NR |
| DECISION+2 Canada Legare, 2012^32^ | To train family physicians in shared decision-making to reduce the overuse of antibiotics in acute respiratory infections | DECISION+2 consisted of a 2-hour online tutorial followed by a 2-hour on-site interactive workshop Online self-tutorial comprised of six modules.1. Introduction 2 Diagnostic probabilities. 3 Treatment. 4. Effective communication of risk and benefits. 5.Promoting active patient participation Interactive workshop consists of- • estimating the diagnostic probabilities for ARTI• describe available therapeutic options and their risks and benefits for treating ARTI • effective communication strategies to share risks and benefits of the antibiotic options • identify the patients’ values and preferences and involving them in decision making to use an antibiotic • use decision support tools(available in the consultation office) that promote shared decision-making. | Department of Family Medicine and Emergency Medicine at Université Laval in 6 regions of Quebec. | Online and interactive workshop |  | Two-hour online tutorial followed by a 2-hour on-site interactive workshop | As part of the intervention, research assistants verified that the decision aids were available in each of the walk-in consultation rooms in all of the family practice teaching units in the intervention arm |
| Electronic health record–based clinical decision support (EHR CDS) Canada Chung, 2017^14^ | To deliver ASP recommendations at the point-of-care through the existing medical record systems | perspectives of antimicrobial stewardship and EHR CDS-Not explicitly reported | AMS study team | NR | NR | NR | NR |
| EQUIP study UK Francis, 2013^21^ | To provide interactive booklet, ‘When should I worry’ as a take home resource, which contained information on symptoms of RTIs, usual course of illness, antibiotics, self-management, and when to seek help. | An interactive booklet, ‘When should I worry’, during consultations with recruited patients with RTIs and their parent and to provide it as a take home resource. As part of development of the intervention, the clinicians were provided with online training describing the content and aims of the booklet, encouraging its use within consultations, and promoting good communication. The training included information and videos providing examples of tasks to be completed during consultations, and took approximately 30 minutes to complete | Study team | NR however available online | The training included information and videos providing examples of tasks to be completed during consultations and took approximately 30 minutes to complete. Both the booklet and training are available online at www.whenshouldiworry.com | The booklet was developed through a multistage process | The booklet was developed through a multistage process |
| ERNIE2 trial- POC CRP Belgium Lemiengre, 2018^34^ | To reduce antibiotic prescribing in children with acute non-severe infections in primary care in comparison to usual care | POC CRP test | Study team | Face to face | NR | NR | NR |
| GAPS Australia Avent, 2024^7^ | To reduce antibiotic prescribing for ARIs | 1. Poster on Practice Antibiotic Prescribing Policy consisted of displaying a poster-sized prescribing policy in the GPs waiting room and/or examination room. 2. Patient information leaflet provided information to the patient about inappropriate use of antibiotics the potential harmful effects of antibiotics. 3. Online communication training package was offered in combination with background information on the problem of AMR in primary care. 4. Delayed antibiotic prescribing consisted of advice to the patient to only fill the prescription at a pharmacy after a few days if symptoms were not starting to settle or become more severe. 5. Patient Decision Aid is a brief graphical laminated summary of evidence for the management of a number of ARI conditions. 6. The CRP test | Research coordinators who have been trained in the use of the interventions. | Face to face and online | 6 months | NR | NR |
| GRACE INTRO multi-Country Anthierens, 2015^5^ | To reduce antibiotic prescribing for adults with acute RTIs in Belgium, England, the Netherlands, Poland, Spain and Wales | All clinicians in the three intervention arms received a web-based intervention. The intervention consisted of three modules; an introduction, training on CRP and training in communication skills [including use of a patient booklet]. | Study team | Web based | NR | NR | Training was developed through piloting intervention materials with clinicians in all participating countries |
| GRACE/INTRO multi-country  Yardley, 2013^59^ | To reduce antibiotic prescribing in primary care | The web-based training consisted of a single session on existing successful theory-based interventions to reduce antibiotic prescribing in primary care. It comprised three sections: an introduction, a module providing training in using a CRP test, and a module providing training in communication skills and use of a patient booklet. the GRACE/INTRO training also illustrated (using videos) how a patient booklet could be used in the consultation to address specific patient concerns; | Study team | Web based | NR | The content of the patient booklet drew on previously validated content that addressed perceptions of symptoms and antibiotics based on the extended Common-Sense Model, which describes the dimensions of symptoms salient to patients (i.e., identity, cause, duration, severity of consequences, and potential for control or cure) as well as the salient dimensions of medication (i.e., perceived need and potential for harm). | The materials were piloted in every country and modified according to feedback from interviews with health professionals and patients in each country, allowing small between-country differences in the website where this seemed advisable |
| HAPPY AUDIT Spain Llor, 2014^38^ | To strengthen the surveillance of respiratory tract infections in primary health care | Partial intervention consisted of meetings with the GPs including prescriber feedback based on the results from the first registration, training courses on the diagnosis and treatment of respiratory tract infections, and review of guidelines on respiratory tract infections. The full intervention will include: • Training course on appropriate use of antibiotics for RTIs • Clinical guidelines including recommendations for diagnosis and treatment of RTIs • Posters for doctors' waiting rooms, focusing on the appropriate use of antibiotics, and targeting all patients visiting practice • Brochures and handouts to patients about prudent use of antibiotics • Point of care (POC) rapid tests: Strep A and CRP • Training in use and interpretation of results from POC rapid tests. | Health Alliance for Prudent Prescribing, Yield and Use of Antimicrobial Drugs in the Treatment of Respiratory Tract Infections (HAPPY AUDIT) project, a study financed by the European Commission. | Offline posters and brochures, face to face POC rapid test | NR | NR | Feedback forms |
| HAPPY AUDIT Spain Llor, 2015^39^ | To strengthen the surveillance of RTIs in primary health care through the development of intervention programmes targeting GPs and changing people’ s habits towards prudent use of antimicrobial agents | The follow-up intervention activities will comprise workshops, clinical skills courses, reminders and clinical training, as well as interdisciplinary training courses. The intervention will start after the first registration period and it will include the following activities: • Training course on appropriate use of antibiotics for RTIs • Clinical guidelines including recommendations for diagnosis and treatment of RTIs • Posters for doctors' waiting rooms, • Brochures and handouts to patients about prudent use of antibiotics • Point of care (POC) rapid tests •Training in use and interpretation of results from POC | Researcher associated with European Commission | Remotely- includes workshop, poster, brochure to hand over | NR | NR | NR |
| HAPPY AUDIT Multi-country Bjerrum, 2011^9^ | Contributing to the battle against antibiotic resistance through quality improvement of GPs' diagnosis and treatment of RTIs through development of intervention programmes targeting GPs, parents of young children and healthy adults. | Shortly after the first registration the GPs were invited to follow-up meetings where they received individual prescriber feedback and identified potential quality problems. Afterwards, they were offered an intervention programme that included the following elements:• Training course on appropriate use of antibiotics for RTIs • Clinical guidelines with recommendations for diagnosis and treatment of RTIs.• Posters for waiting rooms, focusing on the appropriate use of antibiotics• Brochures and handouts to patients about prudent use of antibiotics• Access to Point of care (POC) tests: Strep A and C Reactive Protein (CRP)• Training in use and interpretation of POC tests | 15 Govt partners from 9 countries provided the intervention to the GPs which comprise workshops, clinical skills courses, reminders and clinical training, as well as interdisciplinary training courses. | NR | Once, after the first registration, | NR | NR |
| Parent–child dyads USA Goggin, 2022^24^ | To reduce inappropriate antibiotic prescribing (i.e., number of patients receiving an inappropriate prescription/number of patients in arm). I | Intervention consisted of- 1. 90s video explain the antibiotic prescribing strategies while emphasising the risk of side effects and resistant organism. 2. Parent educational trifold brochure provided ‘gain-framed’ information about when antibiotics are and are not necessary and the risks involved in taking antibiotics. 3.The study physicians provided a 20min, in-person general antibiotic education training (on diagnosis, treatment, pros and cons of antibiotics, impact of inappropriate use, CDC guidelines, antibiotic misuse and viewing/discussing the 90s parent video). 4. The in-person, 50min communication skills training included viewing/discussing motivational role model videos. | Study’s behavioural psychologist (KG) and other researchers | Mix of videos information materials at the primary care and in person training | 90s video and patient information material at primary care, 20min in-person general antibiotic education training on diagnosis and treatment of ARTI, in-person, 50min communication skills training. | We tailored all parent materials to highlight the gains of not using antibiotics (e.g., staying safe from side effects) that might increase parents’ comfort with not getting an antibiotic prescription for their child. | NR |
| POC CRP UK Huddy, 2016^29^ | NR | Two presentations preceded the workshop; the first outlined current evidence for POC CRP including economic analyses, and the second presented the emergent theme analysis from the interview study. Facilitated discussion then covered barriers to adoption, impact of adoption and adoption scenarios. | NR | NR | NR | NR | NR |
| POC CRP The Netherlands Peters, 2013^48^ | To diagnose and monitor bacterial infections | POC CRP test | NR | Face to face at medical service office or at the patient’s bedside. | NR | NR | NR |
| RAAK (RAtional Antibiotic use Kids) intervention The Netherlands Dekker, 2019^17^ | To reduce antibiotic prescribing for children with RTI. | The online training for GPs consisted of (i) general background about the relevance of prudent antibiotic use and information about antibiotic-related problems; (ii) child-specific information including assessment of disease severity, risk factors, signs and symptoms, and the first and second choice antibiotic treatment advised; and (iii) training in enhanced communication skills supported by videos of consultation techniques. The information booklet for parents contained the following information using text and pictograms: epidemiology of RTIs, their predominantly viral cause, self-limiting prognosis, rationale to withhold antibiotics, and antibiotic-related problems, including bacterial resistance. Additionally, the booklet explained self-management strategies for their child and signs and symptoms of when to consult the GP. | Study team | Online | NR | NR |  |
| RTI CPR UK Hounkpatin, 2021^28^ | To predict outcomes of RTIs and help clinicians identify patients who may or may not benefit from antibiotics. | CPRs for RTIs | Local Clinical Research Networks (LCRNs; Wessex and West of England) | NR | NR | NR | NR |
| STAR program  UK Butler, 2012^10^ | To reduce antibiotic dispensing | A combination of various learning methods and topics | Study team | Online-Anywhere | Flexible- at own pace | NR | NR |
| TARGET Antibiotics Toolkit UK Jones, 2017^31^ | To help prescribers and commissioning organizations to increase responsible antimicrobial prescribing in the primary care setting | ARGET Antibiotics Toolkit: comprised of an interactive workshop presentation, patient leaflets (Treating Your Infection), audit toolkits, National antibiotic management guidance, training resources, resources for clinical and waiting areas and a self-assessment checklist | Public Health England with the Royal College of General Practitioners (RCGP) and other professional societies | TARGET workshops given by 10 trainers involved 56 GP practices with 318 primary care staff (including receptionists, practice managers and other non-prescribing staff) were conducted across England as part of a wider evaluation where all practice staffs were invited to take part in the workshop to encourage a whole practice approach to antimicrobial stewardship (AMS). | Trained staff delivered the 1 h workshop covering AMR, guidance, how to optimize antibiotic prescribing, use of resources in the Toolkit, reflection on their own antibiotic prescribing data and some action planning. | NR | Workshop participants completed a fivepoint Likert scale evaluation form immediately after each workshop to assess its effectiveness. |
| TARGET TYI UK Ashiru-Oredope, 2020^6^ | To use during consultations and also take-home information for patients on the usual duration of common self-limiting respiratory infections, including how to self-care, and when to seek further advice | TARGET TYI-RTI community pharmacy leaflet and webinar-based AMS training | Study team | Face to face and webinar | NR | The TARGET TYI-RTI leaflet was adapted and launched for community pharmacy teams in 2015 | The TARGET “treating your infection—respiratory tract infection” (TYI-RTI) leaflet is one evaluated resource already in use within general practice, |
| TARGET TYI (Version 8) UK Eley, 2020^19^ | Designed to be used with patients who are experiencing self-limiting upper RTIs and supports implementation of recommendations in the NICE guidelines on processes for antimicrobial stewardship, behaviour change for antimicrobial stewardship and antibiotic prescribing for RTIs | TARGET antibiotics toolkit and TARGET Treating Your Infection leaflet | Hosted on the Royal College of General Practitioners (RCGP) website, the TARGET toolkit is freely available to all primary care health professionals. | Available on website | NR | NR | The TYI-RTI leaflet is widely used during patient consultations to facilitate conversation about treatment choice |
| The PACE UK Francis, 2020^22^ | To help guide initial antibiotic prescribing decisions for their AECOPD. | Study-specific training, which included guidance for the clinical prescribers on interpreting CRP results in the context of AECOPD. | Study team | Face to face | NR | NR | NR |
| The pharmacy AMS intervention (PAMSI) UK Allison, 2020^3^ | To identify gaps in community pharmacy staff’s capability, opportunity and motivation (COM) to provide AMS advice; and ideas developed at an Information Design and Architecture in Persuasive Pharmacy Space: combating AMR (IDAPPS) workshop | i) A 20 min educational webinar to give pharmacy staff the capability and motivation to check antibiotic appropriateness and provide selfcare and adherence advice to patients/carers collecting antibiotics. ii)An Antibiotic Checklist to give pharmacy staff the capability and opportunity to check antibiotic appropriateness and provide self-care and adherence advice tailored to the individual patient and antibiotic. The A5 folded paper Antibiotic Checklist was designed to fit into the prescription baskets used by most pharmacies and followed the antibiotic prescription’s journey from hand-in to dispensing. (iii) Other patient and staff AMS reinforcing materials, which included posters, shelf signs, counter mats and prescription bag stickers. (iv) Feedback to pharmacy staff about percentage of Antibiotic Checklist completion in their pharmacy and follow-up data from pharmacy users after visiting the pharmacy. | Researchers provided the intervention based on the contents of the Royal Pharmaceutical Society’s AMS checklist | NR | NR | NR | The intervention was development over three workshops through 2018–19, discussed at the second workshop, finalized and then evaluated in community pharmacies within one locality. |
| The Safety Program USA Agency for Healthcare Research and Quality, 2022^2^ | To consider whether antibiotics were indicated, the best empiric antibiotic choices if they were indicated, the need for additional testing such as cultures, evaluation of the patient after the antibiotics are started, and appropriate durations of therapy. | The use of live (and recorded) webinars, narrated presentations, one-page documents and pocket guides (for quick access to diagnosis, management, treatment), commitment posters, patient handouts (ambulatory cohort only), and other tools. The live webinar series, in office hours, addressed aspects of development and sustainability of AS activities, cultural and behavioural drivers of antibiotic decision making, and reviews of best practices in the diagnosis and treatment of patients presenting with common infections. The presentations on management of common infectious diseases were organized according to the Four Moments of Antibiotic Decision-Making Framework. | AMS study team | Live and recorded webinars, pocket cards, information materials | Series of webinars | The educational materials and the Four Moments of Antibiotic Decision Making were tailored to each practice setting. | Pilot period over 1 yr. where the study team obtained feedback forms |
| VISON UK Gulliford, 2014^26^ | To adopt either a no-prescribing or a delayed-prescribing approach during consultations with adults with acute respiratory tract infections | The decision support tools were installed remotely at the intervention arm practices and delivered during consultations through a system known as DXS Point-of Care. The decision support tools provided information for education and decision support (including a summary of antibiotic prescribing recommendations), a single-sided patient information sheet, a summary of research evidence concerning antibiotic prescribing strategies. | Clinical Practice Research Datalink (CPRD) and researchers from King’s College London | Remotely at the intervention arm practices and delivered during consultations through a system known as DXS Point-of Care, | NR | NR | NR |

# Appendix 5: Barriers, facilitators, and corresponding intervention types

|  | **Barrier** | **Facilitators** | **Theoretically Linked Intervention Types**  *(Based on the Behaviour Change Wheel)* |
| --- | --- | --- | --- |
|  | **Healthcare Professionals** | | |
| **Capability** | Limited knowledge of AMR and antibiotics^1,6,7,14,31,55^  Lack of awareness of prescribing patterns^14^  HCPs’ lack of awareness of the available interventions^28,31,41,52,54,55^  Some pharmacists were not aware of the term antimicrobial stewardship.^55^  HCPs were not always aware of computer delivered prompts and clinical prediction rules (CPRs) developed to reduce unnecessary prescribing, and self-help guides ^28,41,55^.  Forgetfulness^8,18,19^  Lack of skills to use the CRP equipment^54^  Inability to persuade patients that no antibiotic is needed^6,52^ | Awareness campaigns targeting both professionals ^23,31,45^  Understanding of threat and impact of AMR^1,22,31^  GPs knew that too many antibiotics are prescribed ^7^.  Pharmacists knowing what self-care advice to give ^6^  Communications trainings,^57^ ^21^.  Training healthcare professionals to perform point-of-care CRP tests^18,29^ | Education  Training  Environmental restructuring  Modelling  Enablement |
| **Opportunity** | Lack of time^1,5-7,14,18,19,22,23,28,29,31,41,50,52,54^. For example, many reported lack of time to give advice to patients^6,7,19^, many felt that electronic tools take too long to use^1^ and some reported finding it challenging to add CRP point-of-care testing to the usual consultation time^18,28^.  CRP point-of-care testing impact on the workflow and workload.^22,29^  Electronic medical record-based intervention was difficult to use, make consultation cumbersome or too long.^1^  Financial challenges relating to CRP point-of-care testing.^18,22,29^  Need for refrigerator for CRP cartridges.^22^.  For digital interventions, the need to keep devices clean and potential theft.^52^  Differences in how materials were provided to patients.^21^  Mismatch in clinician’s treatment plan and booklet message.^21^  Too much information on materials.^55^  Running out of materials.^58^  Printing costs.^8,19,31^  Pharmacists felt self-help guides with too much information would not engage readers.^55^  Lack of internet.^8^  Perceived pressure from patients and other stakeholders (such as parents or carers).^7,14,51^  The need to support another healthcare professional’s prescribing decision was also a barrier to appropriate behaviour.^14^  Lack of clarity regarding who will be responsible to take actions regarding antimicrobial stewardship.^31^  Lack of monitoring - Many healthcare professionals reported not monitoring the effect of TARGET and therefore it is unclear if it is valuable and some were not sure whether they had been displayed in their practice.^31^ Lack of monitoring was said to downgrade the importance of antimicrobial stewardship programs.^58^ | Guidelines on antibiotic prescription (Poss Dooering 2020, Eley 2018).  Interventions to educate patients - posters, printed decision aids, leaflets, booklets and videos (Ackerman 2013, Allison 2020, Anthierens 2015, Avent 2024, Eley 2020, Ashiru-Oredope 2020_Improving management of, Francis 2013, Jones 2018, Tonna 2020, Biezen 2021, Wei 2019, Poss-Doering 2020, Tonkin-Crine 2023, Poss-Doering 2020_converting habits).  Interventions to help identify risk of bacterial infection (CRP point-of care testing and clinical prediction rules). ^5,7,18,22,28,29,41,54^  Accessibility of intervention use.^18,22,29,41,54^ For example Providing CRP point of care testing machine in consulting room could also facilitate use^18^ as well as the portability of the machine.^22^  Aesthetically appealing printed materials.^8^  Providing a variety of tools so that clinicians can choose what fits their communication style or patient needs.^7^  Family physicians requested to be paid for informing patients on why no antibiotics is being prescribed for them because it is time consuming.^23^  Early engagement with stakeholders is also important to enhance uptake of electronic health record clinical decision systems.^14^ | Environmental restructuring  Restriction  Enablement  Modelling  Training |
| **Motivation** | Lack of confidence/trust/belief in the usefulness of intervention or believing that an intervention provides no added value also serve as barriers to their use.^1,5,7,18^ For example, some did not use CRP because they do not believe that CRP tests will have any impact on their clinical decision making.^7^. In some cases, general practitioners were less motivated to use CRP point-of-care testing after being frustrated due to having too many error messages from the machine.^18^ Some believe that too many rests are already being used by doctors.^7^ Some clinicians believe that what patients say and the reality are different and the subjective nature of the questions in some tools such as Centor Criteria and FeverPAIN could lead to inaccurate assessments.^28^  Many clinicians tend not to use the test if they have already decided to prescribe antibiotics.^5^  Some clinicians do not use prompts because they felt they do not need them since they were already working in line with the guidelines.^41^  Some were also concerned that the use of digital devices to educate patients are not compatible with their professional values.^52^  Some believe that over prescription is not an issue in their site.^1^  Many HCPs are not aware of their prescribing patterns.^14^  There is a belief that education on appropriate prescribing is not likely to change longstanding prescribing habits.^1^  Some healthcare professionals believe that patients want antibiotics and will not be satisfied if they do not get them.^1,18,21,22,29,31,51^  Desire to satisfy patients due to the business nature of practices and fear of losing patients as patients are likely to go somewhere else if they are not satisfied.^7,18^  Having emotional concerns and guilt when they do not administer a treatment or when they recommend non-prescription medicinal products which patients will have to pay for.^51^  Some physicians believe delayed prescribing and rescue packs inappropriately shift responsibility of clinical decisions to patients and some patients find it difficult to understand when to use the antibiotics.^22,51^ Also, patients may use antibiotic immediately rather than wait.^54^  Some general practitioners are concerned that reducing antimicrobial prescribing would result in increase in hospital admissions so they prescribe antimicrobials to avoid missing infections or avoid patient’s conditions worsening.^22,31^  Fear of litigation.^7,22^  Some general practitioners, particularly those who are more experienced felt nurses, pharmacists or healthcare assistants are best suited to perform CRP point-of-care tests.^18^  The desire to limit the use of paper may be a barrier to the use of printed materials.^8^  Some clinicians believe patients want to be educated directly rather than engage with health-related information.^50^  Some HCPs reported that patients do not appear to read the provided brochure or understand the information.^1^  In the case of electronic health record decision support systems, alert fatigue was a common issue, they were disruptive to workflow, and the alerts are ignored.^14^  Some believe that using clinical prediction rule may impact negatively on consultation as the clinician would need to focus on the computer rather than the patient at some point.^28^ | HCPs would be more likely to use clinical prediction rules if there are strong evidence supporting its effectiveness and it has been adequately validated and tested in the primary care population.^28^  Showing HCPs the data on their prescribing is thought to be potentially a useful strategy to motivate them to change practice as many are not aware of their prescribing patterns.^14^  General practitioners believe patients appreciate delayed prescribing as it provides patients with a safety net and prevents patients from getting worse.^7,22^  Appropriate reimbursement for CRP POC testing could be useful, although careful consideration is required since inadequate reimbursement systems may encourage inappropriate use or overuse.^29^  In a study that used antibiotic Champions, it was reported that those who volunteered and had dedicated time for antimicrobial stewardship were more enthusiastic and engaged better with the intervention materials compared to those who were nominated.^54^  Some also reported being very restrictive and reluctant to prescribe antibiotics even before taking part in the study^51^ or having pre-existing passion for appropriate antibiotic prescribing.^7^  Many pharmacists believe that they play a key role in controlling antibiotic use and giving advice for common infection is important for them.^6^ | Education  Persuasion  Modelling/champions  Enablement  Incentivisation  Coercion  Environmental restructuring  Training |
| **Patients** | | | |
| **Capability** | Limited knowledge about antimicrobial resistance, antibiotics and self-care.^3,7,8,19,21,22,45^  Lack of understanding of AMR transmission.^45^  In some cases, patients felt the information provided in the materials were things they knew already and there are also issues with having conflicting messages from clinicians.^21^  Healthcare professionals also noted that some patients may not have technology skills necessary to electronic/online materials.^8^  Some general practitioners believe younger population know more about antibiotic use than the older population.^7^ | Knowledge about antimicrobial resistance, antibiotics and self-care.^3,7,8,18,21,22,45^  Awareness campaigns targeting the public^23,31,45^ | Education  Training  Environmental restructuring  Modelling  Enablement |
| **Opportunity** | some may view the booklet as a way to discourage them from seeing the doctor.^21^  Some childcare centre regulations allow children with certain symptoms go back if they are on antibiotics, this is thought to be one of the reasons parents often demand antibiotics.^7^  When information was provided on tablets in waiting area, patients were concerned about risk of infection.^50,52^ | Tools, such as posters, leaflets and decision aids, which are used by healthcare professionals during consultation are also useful to improve patient knowledge.^3,7,8,19,21,45^  Clinicians reported that CRP is a way of educating patients for the future and gave patients confidence.^18,22^  Access to self-care advise/pharmacy, facilities to self-care at home, information on self-care and when to get help and having the time for respiratory tract infections to get better on their own are necessary for appropriate antibiotic behaviour.^19^  Patients suggested having information sheets, posters and booklets in the general practice waiting rooms and pharmacies would be useful.^8^ | Environmental restructuring  Restriction  Enablement  Modelling  Training |
| **Motivation** | Some patients consult with a prior determination that they need antibiotics and are disappointed when they do not receive a prescription, especially when they feel they have not received thorough examination or sufficient information.^20,21^  Patient’s trust in the healthcare professionals seems to encourage them to follow the professionals advise.^45^  Some patients may find it difficult to stand up against healthcare professionals’ suggestion even if they feel it is wrong.^52^ | Desire not to take antibiotics unnecessarily.^22^ | Education  Persuasion  Modelling/champions  Enablement  Incentivisation  Coercion  Environmental restructuring  Training |

# References

1. Ackerman SL, Gonzales R, Stahl MS, Metlay JP. One size does not fit all: evaluating an intervention to reduce antibiotic prescribing for acute bronchitis. *BMC Health Services Research* 2013; **13**: 462.

2. Chicago JHMaNatUo. Agency For Healthcare and Quality Safety Program for Improving Antibiotic Use. Rockville Agency for Healthcare Research and Quality, 2022.

3. Allison R, Chapman S, Howard P, et al. Feasibility of a community pharmacy antimicrobial stewardship intervention (PAMSI): An innovative approach to improve patients' understanding of their antibiotics. *JAC-Antimicrobial Resistance* 2020; **2**(4).

4. Andreeva E, Melbye H. Usefulness of C-reactive protein testing in acute cough/respiratory tract infection: an open cluster-randomized clinical trial with C-reactive protein testing in the intervention group. *BMC Family Practice* 2014; **15**: 80.

5. Anthierens S, Tonkin-Crine S, Cals JW, et al. Clinicians' views and experiences of interventions to enhance the quality of antibiotic prescribing for acute respiratory tract infections. *Journal of General Internal Medicine* 2015; **30**(4): 408-16.

6. Ashiru-Oredope D, Doble A, Thornley T, et al. Improving Management of Respiratory Tract Infections in Community Pharmacies and Promoting Antimicrobial Stewardship: A Cluster Randomised Control Trial with a Self-Report Behavioural Questionnaire and Process Evaluation. *Pharmacy : A Journal Of Pharmacy Education And Practice* 2020; **8**(1): 19.

7. Avent ML, Hall L, van Driel M, et al. Reducing antibiotic prescribing in general practice in Australia: a cluster randomised controlled trial of a multimodal intervention. *Australian Journal of Primary Health* 2024; **30**: 1.

8. Biezen R, Manski-Nankervis JA, Somasundaram K, Buising K. Shared decision support for patients. An antimicrobial stewardship strategy to promote appropriate antibiotics use in primary care. *Australian Journal of Primary Health* 2021; **27**(4): vii.

9. Bjerrum L, Munck A, Gahrn-Hansen B, et al. Health Alliance for prudent antibiotic prescribing in patients with respiratory tract infections (HAPPY AUDIT) -impact of a non-randomised multifaceted intervention programme. *BMC family practice* 2011; **12**(1).

10. Butler CC, Simpson SA, Dunstan F, et al. Effectiveness of multifaceted educational programme to reduce antibiotic dispensing in primary care: practice based randomised controlled trial. *BMJ* 2012; **344**: d8173.

11. Cals JWL, Schot MJC, de Jong SAM, Dinant G-J, Hopstaken RM. Point-of-Care C-Reactive Protein Testing and Antibiotic Prescribing for Respiratory Tract Infections: A Randomized Controlled Trial. *Annals of family medicine* 2010; **8**(2).

12. Cals JW, de Bock L, Beckers PJ, et al. Enhanced communication skills and C-reactive protein point-of-care testing for respiratory tract infection: 3.5-year follow-up of a cluster randomized trial. *Annals of Family Medicine* 2013; **11**(2): 157-64.

13. Chiswell E, Hampton D, Okoli CTC. Effect of Patient and Provider Education on Antibiotic Overuse for Respiratory Tract Infections. *Journal for Healthcare Quality* 2019; **41**(3): e13-e20.

14. Chung P, Sc, lyn J, Dayan PS, Mistry RD. Working at the intersection of context, culture, and technology: Provider perspectives on antimicrobial stewardship in the emergency department using electronic health record clinical decision support. *American Journal of Infection Control* 2017; **45**(11): 1198-202.

15. Cross ELA, Sivyer K, Islam J, et al. Adaptation and implementation of the ARK (Antibiotic Review Kit) intervention to safely and substantially reduce antibiotic use in hospitals: a feasibility study. *Journal of Hospital Infection* 2019; **103**(3): 268-75.

16. Dekker ARJ, Verheij TJM, Broekhuizen BDL, et al. Effectiveness of general practitioner online training and an information booklet for parents on antibiotic prescribing for children with respiratory tract infection in primary care: a cluster randomized controlled trial. *Journal of Antimicrobial Chemotherapy* 2018; **73**(5): 1416-22.

17. Dekker ARJ, van der Velden AW, Luijken J, Verheij TJM, van Giessen A. Cost-effectiveness analysis of a GP- and parent-directed intervention to reduce antibiotic prescribing for children with respiratory tract infections in primary care. *Journal of Antimicrobial Chemotherapy* 2019; **74**(4): 1137-42.

18. Eley CV, Sharma A, Lecky DM, Lee H, McNulty CAM. Qualitative study to explore the views of general practice staff on the use of point-of-care C reactive protein testing for the management of lower respiratory tract infections in routine general practice in England. *BMJ Open* 2018; **8**(10): e023925.

19. Eley CV, Lecky DM, Hayes CV, McNulty CA. Is sharing the TARGET respiratory tract infection leaflet feasible in routine general practice to improve patient education and appropriate antibiotic use? A mixed methods study in England with patients and healthcare professionals. *Journal of Infection Prevention* 2020; **21**(3): 97-107.

20. Forrest CL, Verzone A. Antibiotic stewardship: Improving patient-centered right care in urgent care using a shared decision aid and 5 Ds tool. *Journal of the American Association of Nurse Practitioners* 2020; **33**(12): 1265-72.

21. Francis NA, Phillips R, Wood F, Hood K, Simpson S, Butler CC. Parents' and clinicians' views of an interactive booklet about respiratory tract infections in children: a qualitative process evaluation of the EQUIP randomised controlled trial. *BMC Family Practice* 2013; **14**: 182.

22. Francis NA, Gillespie D, White P, et al. C-reactive protein point-of-care testing for safely reducing antibiotics for acute exacerbations of chronic obstructive pulmonary disease: the PACE RCT. *Health technology assessment (Winchester, England)* 2020; **24**(15).

23. Giry M, Pulcini C, Rabaud C, Boivin JM, Mauffrey V, Birge J. Acceptability of antibiotic stewardship measures in primary care. *Medecine et Maladies Infectieuses* 2016; **46**(6): 276-84.

24. Goggin K, Hurley EA, Lee BR, et al. Let's Talk About Antibiotics: a randomised trial of two interventions to reduce antibiotic misuse. *BMJ Open* 2022; **12**(11): e049258.

25. Gonzales R, Anderer T, McCulloch CE, et al. A cluster randomized trial of decision support strategies for reducing antibiotic use in acute bronchitis. *JAMA Internal Medicine* 2013; **173**(4): 267-73.

26. Gulliford MC, van Staa T, Dregan A, et al. Electronic health records for intervention research: a cluster randomized trial to reduce antibiotic prescribing in primary care (eCRT study). *Annals of Family Medicine* 2014; **12**(4): 344-51.

27. Hernandez-Santiago V, Marwick C, Patton A, Davey P, Donnan PT, Guthrie B. Time series analysis of the impact of an intervention in Tayside, Scotland to reduce primary care broad-spectrum antimicrobial use. *The Journal of antimicrobial chemotherapy* 2015; **70**(8).

28. Hounkpatin HO, Woods C, Lown M, Stuart B, Leydon GM. Understanding GPs' views and experiences of using clinical prediction rules in the management of respiratory infections: a qualitative study. *Bjgp Open* 2021; **5**(4).

29. Huddy JR, Ni MZ, Barlow J, Majeed A, Hanna GB. Point-of-care C reactive protein for the diagnosis of lower respiratory tract infection in NHS primary care: a qualitative study of barriers and facilitators to adoption. *BMJ Open* 2016; **6**(3): e009959.

30. Jenkins TC, Irwin A, Coombs L, et al. Effects of clinical pathways for common outpatient infections on antibiotic prescribing. *American Journal of Medicine* 2013; **126**(4): 327-35.e12.

31. Jones LF, Hawking MKD, Owens R, et al. An evaluation of the TARGET (Treat Antibiotics Responsibly; Guidance, Education, Tools) Antibiotics Toolkit to improve antimicrobial stewardship in primary care-is it fit for purpose? *Family Practice* 2018; **35**(4): 461-7.

32. Legare F, Labrecque M, Cauchon M, Castel J, Turcotte S, Grimshaw J. Training family physicians in shared decision-making to reduce the overuse of antibiotics in acute respiratory infections: a cluster randomized trial. *CMAJ Canadian Medical Association Journal* 2012; **184**(13): E726-34.

33. Lemiengre MB, Verbakel JY, Colman R, et al. Reducing inappropriate antibiotic prescribing for children in primary care: a cluster randomised controlled trial of two interventions. *British Journal of General Practice* 2018; **68**(668): e204-e10.

34. Lemiengre MB, Verbakel JY, Colman R, et al. Point-of-care CRP matters: normal CRP levels reduce immediate antibiotic prescribing for acutely ill children in primary care: a cluster randomized controlled trial. *Scandinavian journal of primary health care* 2018; **36**(4).

35. Likopa Z, Kivite-Urtane A, Silina V, Pavare J. Impact of educational training and C-reactive protein point-of-care testing on antibiotic prescribing in rural and urban family physician practices in Latvia: a randomised controlled intervention study. *BMC pediatrics* 2022; **22**(1).

36. Little P, Stuart B, Francis N, et al. Antibiotic Prescribing for Acute Respiratory Tract Infections 12 Months After Communication and CRP Training: A Randomized Trial. *Annals of Family Medicine* 2019; **17**(2): 125-32.

37. Little P, Stuart B, Francis N, et al. Effects of internet-based training on antibiotic prescribing rates for acute respiratory-tract infections: a multinational, cluster, randomised, factorial, controlled trial. *Lancet* 2013; **382**(9899): 1175-82.

38. Llor C, Bjerrum L, Munck A, et al. Access to point-of-care tests reduces the prescription of antibiotics among antibiotic-requesting subjects with respiratory tract infections. *Respiratory Care* 2014; **59**(12): 1918-23.

39. Llor C, Monedero MJ, Garcia G, Arranz J, Cots JM, Bjerrum L. Interventions to improve adherence to first-line antibiotics in respiratory tract infections. The impact depends on the intensity of the intervention. *European Journal of General Practice* 2015; **21**(1): 12-8.

40. Madaras-Kelly K, Hostler CJ, Townsend ML, et al. Impact of Implementation of the Core Elements of Outpatient Antibiotic Stewardship Within Veterans Health Administration Emergency Departments and Primary Care Clinics on Antibiotic Prescribing and Patient Outcomes. *Clinical infectious diseases : an official publication of the Infectious Diseases Society of America* 2020; **73**(5).

41. McDermott L, Yardley L, Little P, et al. Process evaluation of a point-of-care cluster randomised trial using a computer-delivered intervention to reduce antibiotic prescribing in primary care. *BMC Health Services Research* 2014; **14**: 594.

42. McIsaac WJ, Senthinathan A, Moineddin R, et al. Development and evaluation of a primary care antimicrobial stewardship program (PC-ASP) in Toronto, Ontario, Canada. *Journal of the Association of Medical Microbiology and Infectious Disease Canada = Journal officiel de lAssociation Pour La Microbiologie Medicale et linfectiologie Canada* 2021; **6**(1): 32-48.

43. Meeker D, Knight TK, Friedberg MW, et al. Nudging guideline-concordant antibiotic prescribing: a randomized clinical trial. *JAMA Intern Med* 2014; **174**(3): 425-31.

44. Milos V, Jakobsson U, Westerlund T, Mel, er E, Molstad S, Midlov P. Theory-based interventions to reduce prescription of antibiotics-A randomized controlled trial in Sweden. *Family Practice* 2013; **30**(6): 634-40.

45. Mowbray F, Sivyer K, Santillo M, et al. Patient engagement with antibiotic messaging in secondary care: a qualitative feasibility study of the 'review and revise' experience. *Pilot and feasibility studies* 2020; **6**(1).

46. Muhia CL. Using a Delayed Antibiotic-Prescribing Education Intervention to Prevent Antibiotic Overuse in the Treatment of Respiratory Tract Infections. *NA* 2016; **NA**(NA).

47. Patel D, Ng T, Madani LS, et al. Antibiotic stewardship to reduce inappropriate antibiotic prescribing in integrated academic health-system urgent care clinics. *Infection Control & Hospital Epidemiology* 2022: 1-10.

48. Peters CM, Schouwenaars FM, Haagsma E, Evenhuis HM, Echteld MA. Antibiotic prescribing and C-reactive protein testing for pulmonary infections in patients with intellectual disabilities. *British Journal of General Practice* 2013; **63**(610): e326-30.

49. Kim P, Barbara LW, Robert SM, Blackmore CC. Improving Acute Respiratory Infection Care Through Nurse Phone Care and Academic Detailing of Physicians. *The Journal of the American Board of Family Medicine* 2015; **28**(2): 195.

50. Poss-Doering R, Kuehn L, Kamradt M, et al. Converting habits of antibiotic use for respiratory tract infections in German primary care (CHANGE-3) - process evaluation of a complex intervention. *BMC family practice* 2020; **21**(1).

51. Poss-Doering R, Kamradt M, Stuermlinger A, Glassen K, Kaufmann-Kolle P, Andres E, Wensing M. The complex phenomenon of dysrational antibiotics prescribing decisions in German primary healthcare: a qualitative interview study using dual process theory. *Antimicrobial Resistance & Infection Control* 2020; **9**(1): 6.

52. Poss-Doering R, Kuehn L, Kamradt M, Glassen K, Wensing M. Applying Digital Information Delivery to Convert Habits of Antibiotic Use in Primary Care in Germany: Mixed-Methods Study. *Journal of medical Internet research* 2020; **22**(10).

53. Sloane PD, Zimmerman S, Ward K, et al. A 2-Year Pragmatic Trial of Antibiotic Stewardship in 27 Community Nursing Homes. *J Am Geriatr Soc* 2020; **68**(1): 46-54.

54. Tonkin-Crine S, McLeod M, Borek AJ, et al. Implementing antibiotic stewardship in high-prescribing English general practices: a mixed-methods study. *The British journal of general practice : the journal of the Royal College of General Practitioners* 2023; **73**(728).

55. Tonna AP, Weidmann AE, Sneddon J, Stewart D. Views and experiences of community pharmacy team members on antimicrobial stewardship activities in Scotland: a qualitative study. *International Journal of Clinical Pharmacy* 2020; **42**(5): 1261-9.

56. van Esch TEM, Brabers AEM, Hek K, van Dijk L, Verheij RA, de Jong JD. Does shared decision-making reduce antibiotic prescribing in primary care? *Journal of Antimicrobial Chemotherapy* 2018; **73**(11): 3199-205.

57. Wei X, Zhang Z, Walley JD, et al. Effect of a training and educational intervention for physicians and caregivers on antibiotic prescribing for upper respiratory tract infections in children at primary care facilities in rural China: a cluster-randomised controlled trial. *The Lancet Global Health* 2017; **5**(12): e1258-e67.

58. Wei X, Zhang Z, Hicks JP, et al. Long-term outcomes of an educational intervention to reduce antibiotic prescribing for childhood upper respiratory tract infections in rural China: Follow-up of a cluster-randomised controlled trial. *PLoS Medicine / Public Library of Science* 2019; **16**(2): e1002733.

59. Yardley L, Douglas E, Anthierens S, et al. Evaluation of a web-based intervention to reduce antibiotic prescribing for LRTI in six European countries: quantitative process analysis of the GRACE/INTRO randomised controlled trial. *Implementation Science* 2013; **8**: 134.
